# The Candidate Histocompatibility Locus of a Basal Chordate Encodes Two Highly Polymorphic Proteins

**DOI:** 10.1371/journal.pone.0065980

**Published:** 2013-06-24

**Authors:** Marie L. Nydam, Nikolai Netuschil, Erin Sanders, Adam Langenbacher, Daniel D. Lewis, Daryl A. Taketa, Arumugapradeep Marimuthu, Andrew Y. Gracey, Anthony W. De Tomaso

**Affiliations:** 1 Department of MCD Biology, University of California Santa Barbara, Santa Barbara, California, United States of America; 2 Department of Marine Environmental Biology, University of Southern California, Los Angeles, California, United States of America; Chang Gung University, Taiwan

## Abstract

The basal chordate *Botryllus schlosseri* undergoes a natural transplantation reaction governed by a single, highly polymorphic locus called the *fuhc*. Our initial characterization of this locus suggested it encoded a single gene alternatively spliced into two transcripts: a 555 amino acid–secreted form containing the first half of the gene, and a full-length, 1008 amino acid transmembrane form, with polymorphisms throughout the ectodomain determining outcome. We have now found that the locus encodes two highly polymorphic genes which are separated by a 227 bp intergenic region: first, the secreted form as previously described, and a second gene encoding a 531 amino acid membrane-bound gene containing three extracellular immunoglobulin domains. While northern blotting revealed only these two mRNAs, both PCR and mRNA-seq detect a single capped and polyadenylated transcript that encodes processed forms of both genes linked by the intergenic region, as well as other transcripts in which exons of the two genes are spliced together. These results might suggest that the two genes are expressed as an operon, during which both genes are co-transcribed and then trans-spliced into two separate messages. This type of transcriptional regulation has been described in tunicates previously; however, the membrane-bound gene does not encode a typical Splice Leader (SL) sequence at the 5′ terminus that usually accompanies trans-splicing. Thus, the presence of stable transcripts encoding both genes may suggest a novel mechanism of regulation, or conversely may be rare but stable transcripts in which the two mRNAs are linked due to a small amount of read-through by RNA polymerase. Both genes are highly polymorphic and co-expressed on tissues involved in histocompatibility. In addition, polymorphisms on both genes correlate with outcome, although we have found a case in which it appears that the secreted form may be major allorecognition determinant.

## Introduction

Allorecognition is the ability of an individual to distinguish its own tissues from those of another individual, and examples are found throughout the animal kingdom [1], [2]). The colonial ascidian (Tunicate) *Botryllus schlosseri* undergoes a natural transplantation reaction controlled by a single, highly polymorphic locus called the *fuhc*, for **fu**sion/**h**isto**c**ompatibility) [3]–[5]. This reaction is initiated when terminal portions of the vasculature (called ampullae) come into contact, after which they will either fuse together, forming a vascular and hematopoietic chimera, or reject, an inflammatory reaction which blocks vascular fusion. Fusion and rejection is determined by the *fuhc* with the following rule: two individuals which share one or both *fuhc* alleles are compatible and will fuse, while those sharing neither allele will reject each other. This outcome is clearly due to gene(s) in a single locus, as fusibility segregates in normal Mendelian ratios, even in crosses between wild-type individuals [3]. Finally, the locus is incredibly polymorphic, with most populations showing between 50 to hundreds of specificities, meaning that interactions between unrelated individuals will usually result in a rejection [3].

Tunicates are basal chordates, and this close phylogenetic relationship to the vertebrates, coupled to the commonality of a single locus controlling allorecognition specificity was the basis of our original working hypothesis that the *fuhc* may be the precursor of the jawed vertebrate MHC [3]. *B. schlosseri* is lab-reared, and strains carrying known allorecognition specificities have been derived. Using these resources, we took a forward genetic approach and delineated a ca. 1cM region that segregated with allorecognition outcomes [6]–[7]. Within this locus, we identified three genes involved in allorecognition, including a candidate histocompatibility ligand and two putative receptors [7]–[9]. Initial analysis of this candidate ligand (c*fuhc*) suggested that it was a single, 31 exon gene with two alternative splice variants, one encoding a 555 residue secreted form (exons 1–17), and a second a 1008 residue membrane-bound form (exons 1–14 spliced to exons 18–31). Although the candidate full-length protein contained several immunoglobulin (Ig) domains, it was clearly not related to the vertebrate MHC. In the intervening period, candidate allorecognition molecules in both another ascidian species [10] and a cnidarian [11] have been described, and the genome of a marine sponge (*Amphimedon queenslandica*) with a well-studied allorecognition system has been completed [12]. Surprisingly, there is no conservation among these newly discovered candidate molecules. The cnidarian histocompatibility loci (*alr1* and *alr2*) encode membrane bound molecules with immunoglobulin-like domains that are not homologous to those found in *Botryllus* [11], while sperm/egg interactions in *Ciona intestinalis*, another tunicate, are controlled by a polycystin-related receptor binding to a fibrinogen-like ligand [10]. In turn, homologs of these genes have not been identified in the *A. queenslandica* genome [12]. Even among the vertebrates there is a complete lack of conservation, as studies in jawless fish demonstrate that they have a unique adaptive immune response based on Leucine-Rich-Repeat (LRR) proteins, versus the Ig-based proteins used by the jawed vertebrates [13]. In summary, there is no evolutionary link among multiple allorecognition systems, even between closely related species. Thus the origins of each of these complex recognition systems, from those controlling transplantation specificity in different invertebrate species, to the MHC-based allorecognition that is the center of vertebrate adaptive immunity, are unknown.

Despite disparate evolutionary origins, a unifying characteristic of these systems is that they are all highly polymorphic, with tens to hundreds of allorecognition specificities [14]. Underlying this specificity is both highly precise recognition events as well as mechanisms that would prevent auto-reactivity, i.e., tolerance. How these processes occur in any non-vertebrate organism are not understood, but it may be these features which are conserved, as the generation and maintenance of specificity is not dependent on the nature of the ligands and receptors involved, rather how effector cells are responding to binding events that are occurring at the cell surface [2].

*B. schlosseri* is an excellent model to explore these questions. In addition to its genetic tractability (described above), the allorecognition reaction occurs in a single cell epithelial layer at the tips of a macroscopic extracorporeal vasculature (called ampullae) outside the body that can be directly visualized and manipulated in vivo. In addition, functional assays have been developed for functional studies both in vivo and in vitro [8], [9].

During our recent studies on *fuhc* encoded receptors and ligands we have found that our original characterization of the candidate *fuhc* gene was incorrect [7]. Instead of a single, alternatively spliced gene which made two transcripts (a 555 residue secreted form and a 1008 residue membrane bound form that included most of the secreted ectodomain), we have now found that the candidate locus likely encodes two independent genes separated by a 227bp intergenic region: the secreted gene as originally described, and a smaller, 523 residue membrane bound form. Despite this change, we have found that both genes are highly polymorphic and show evidence of positive selection [15], and that the polymorphisms of both genes correlate with allorecognition outcome. In addition, both are expressed on the tips of the ampullae. We have also found that there are stable intermediate mRNAs that encode both genes, which might be indicative of either a novel transcriptional regulatory event, or a consequence of the tight linkage of the two genes. While in one sense this does not change previous conclusions, characteristics of both genes indicate that our original working hypotheses on how allorecognition specificity is achieved may be more complicated than originally envisioned, as allele specific polymorphisms may be encoded on both a secreted and membrane-bound protein.

## Materials and Methods

### RACE, RT-PCR, qPCR cloning, sequencing, mRNA seq

Total RNA was isolated from whole colony or dissected tissues (as noted) using Nucleo-Spin II columns from Macherey Nagel, and mRNA was isolated using the NEB Magnetic Bead Isolation kit. CAP-trapping was done according to [33]. For RACE and RT-PCR, we used Clontech Advantage 1 or Advantage 2 Taq polymerase and the SMART system following the manufacturers′ recommendations for PCR conditions. cDNA was made using Superscript II from Invitrogen. For nested PCR, we diluted the original PCR reaction 1/200 in water and used 1 uL for the second amplification with new primers. Quantitative PCR analysis was done as described in [9]. Primers for *fuhc* ^tm^ were from exons 4F and 5R, and for *fuhc* ^sec^ were 9F and 10R (below). The *fuhc* ^tm^ sequence has been deposited in GenBank, accession number JX625138.1.

PCR products were isolated using Qiagen columns, then subcloned into the Promega Easy-T vector and transformed using competent DH5a from NEB. Single colonies were picked and inserts amplified using colony PCR and NEB Standard Taq. PCR products were prepared for sequencing using NEB exo/sap, then sent for Sanger sequencing at the UC Berkeley Sequencing Facility. Sequence was analyzed using DNASTAR software. Proteins structure was analyzed using ELM [17]. For mRNA seq, libraries were prepared and sequenced at the USC genome center using kits from Illumina following the manufacturers' instructions. Alignments were done using Bowtie and SMALT, and visualized using Tablet [24].

### Primers used in this study

F is sense, R is antisense with respect to the mRNA.

*fuhc* ^tm^ exon 1F CAAGATCCTACAGGAAGTATCAGC

*fuhc* ^tm^ exon 2R CAAAGTTCCTTTATAGGCTGCAC

*fuhc* ^tm^ exon 4F gtatgggacaacacaggaaattctac

*fuhc* ^tm^ exon 5R gtgacgttttagtccataggatatcag

*fuhc* ^tm^ exon 13R GATACTTGGCTCTCGCCTTGATCTT

*fuhc* ^sec^ exon 3F AAATCTAACGTTCCCTTATTATCTCC

*fuhc* ^sec^ exon 10R TAGCTCCTGGTTCATCGTATAAATATC

*fuhc* ^sec^ exon 9F tactattgagtgtatgaacggtgatgt

*fuhc* ^sec^ exon 10R ttctatcgcccctatatagttttgtaa

*fuhc* ^tm^ exon 9R GTACCTCAAGTACCACACGCCCCAAT

*fuhc* ^sec^ exon 2F gaaatgttgctgaaaatattctgtctt

Overlap F TACGTTGATTGGAACTGTCGACTTGAAGTA

Overlap R TACTTCAAGTCGACaGTTCCA

Intergenic F TCATATCGTCTGTTATTTAGTTTGCTC

Intergenic R AATCAAATTACACCCACATTTATCTGA.

### Northern blotting

Northern blotting was done using standard techniques with ^32^P-labeled probes [35]. For the *fuhc* ^tm^ the probe covered exons 1–13; the probe for *fuhc* ^sec^ was exons 1–14. Blots were done using both total RNA and mRNA with equivalent results.

### In situ hybridization

Probes covered exons 3–10 for *fuhc* ^sec^, and 1–5 of *fuhc* ^tm^. Probes were labeled with DIG or DNP (Boehringer Mannheim). Single *Botryllus* systems were fixed with 4% formaldehyde in 0.1 M MOPS pH 7.5, 0.5 M NaCl for 3 hours and then transferred to methanol. In order to reduce pigmentation, animals were bleached in 6% H_2_O_2_/methanol and then returned to methanol. After rehydration, animals were permeabilized with proteinase K and then post-fixed with 4% formaldehyde. Prehybridization was carried out for 6 hours at 65°C in hybridization buffer (65% formamide, 5X SSC, 1X Denhardt's solution, 0.1% Tween-20, 5 mg/ml torula yeast RNA, 50 ug/ml heparin), followed by hybridization with DIG- and DNP-labeled probes in Hybridization buffer overnight at 65°C. After washing off unbound probes, DIG-labeled probe was detected with an anti-DIG HRP-conjugated antibody (Roche) and TSA Plus Cy3 reagent (Perkin Elmer). The HRP-conjugated anti-DIG antibody was then inactivated with 2% H_2_O_2_ in PBS with 0.1% Triton X-100. DNP-labeled probe was then detected with anti-DNP HRP-conjugated antibody (Perkin Elmer) and TSA Plus Fluorescein reagent (Perkin Elmer). After washing in PBS, specimens were flat-mounted with Vectashield (Vector Labs) and imaged on an Olympus Fluoview 500 confocal microscope.

### Correlation of allorecognition outcome to *fuhc* ^tm^ and *fuhc* ^sec^ polymorphisms

Lab-reared animals were subcloned and paired as described [7], and fusion or rejection visually assessed. For the Santa Barbara animals, we did a round-robin experiment using multiple individuals, and then picked a subset of five which showed nearly equivalent amounts of fusion or rejection when tested against each other (4 fusions and 6 rejections). Naïve subclones of each individual were reared in captivity for one week to ensure they were not pregnant, then RNA isolated and both genes amplified and sequenced. For the HM9aYx1225/SC32e/SC27F, exons 2–14 of fuhc^sec^ and 1–13 of *fuhc* ^tm^ were analyzed. In the experiments using animals from Santa Barbara, exons 2–10 of *fuhc* ^sec^ and exons 1–9 of *fuhc* ^tm^ were analyzed. All cDNA and translated protein sequences from this study can be found in Figure S1.

### Expression of the original candidate *fuhc* gene in 293T cells

Exons 1–27 of the original full-length candidate *fuhc* were fused to the EGFP cDNA, cloned into a MSCV vector and expressed in 293T cells as previously described for fester and uncle fester [8], [9].

## Results

### Initial cloning of mRNAs from the *fuhc* locus

**Figure 1:**
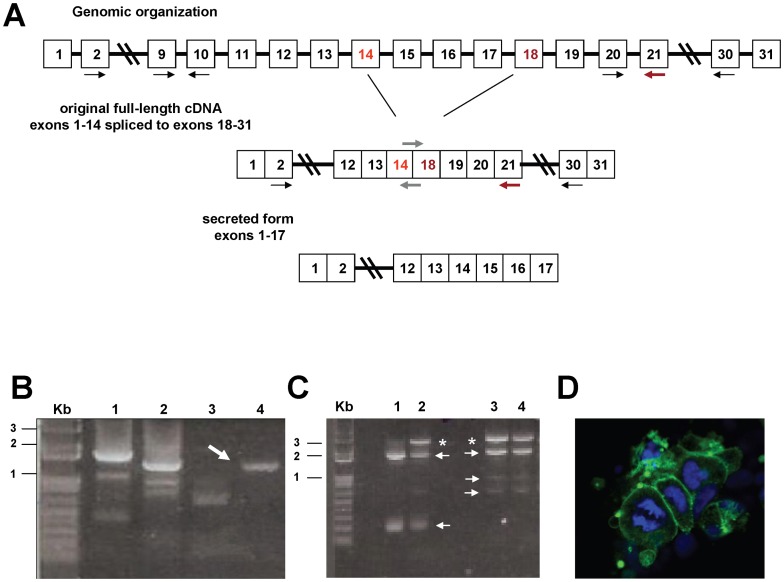
Original characterization of the candidate *fuhc*. **A.** Top shows an illustration of the genomic structure of the *fuhc* locus using our original exon numbering. Middle illustration represents the original full-length cDNA which encoded exons 1–14 spliced to 18–31. The secreted cDNA (exons 1–17) is shown on the bottom. Primers used in panels B and C are represented by arrows. Gray arrows represent primers which spanned the exon 14/18 boundary (see text). The red arrow was used for 5′ RACE shown in panel B. **B.** Initial RACE results using original exon numbering. Sense primers for 3′ RACE: lane 1, sense primer in exon 20; lane 2, sense primer in exon 9. For 5′ RACE, Lane 3 is an amplicon using an antisense primer in exon 10, In lane 4 the antisense primer was in exon 21 (red arrow in panel A; middle). Sequence from the RACE product in Lane 4 (white arrow) was the original evidence for alternative splicing. It should have amplified a 373 bp fragment (to the front of the *fuhc* ^tm^ mRNA), but instead amplified a product that spanned the two genes. **C.** Amplification of the full-length gene from primers in exon 2 and exon 30 from cDNA isolated from 4 wild-type genotypes. The expected 3.2 Kb full-length product is amplified (asterisks), as well as several smaller products which encoded splice variants missing various exons but still linking the two genes (arrows). **D.** Retroviral expression of exons 1–27 of the original transmembrane gene fused to EGFP in mammalian 293T cells. The protein (green) is targeted to the plasma membrane, suggesting it is folding correctly and transiting the secretory pathway normally. Nuclei are counterstained with DAPI.

During positional cloning, we initially amplified the candidate *fuhc* (c*fuhc*) cDNA using two primer sets based on a predicted gene identified within a Fosmid clone. One primer set was in the 5′ region of the predicted cDNA, and another in the 3′ end [7]. Using our original published exon assignments as reference (Figure 1A, top), the first primer set had a sense (S) primer in exon 9, anti-sense (AS) primer in exon 10, while the second primer pair was closer to the 3′ region (S primer in exon 20, AS primer in exon 21). Both amplified a small region (approximately 200 bp) of the predicted mRNA with the correct sequence. Next we used each S primer for a 3′ RACE and each AS primer for a 5′ RACE. For the 3′ RACE, both primers amplified a strong product; however, the most 5′ sense primer (from exon 9) amplified a product that was smaller than the downstream sense primer (located in exon 20). Sequencing of these products revealed two products, the shorter one encoding exons 9–17, and the other exons 21–31 (Figure 1B) [7].

The 5′ RACE gave different results. The product amplified from the anti-sense primer in exon 10 encoded exons 1–10; however, the 5′ RACE from the anti-sense primer in exon 21 amplified a 1.5 Kb product which encoded exons 1–12 linked to exons 19–21 (Figure 1B, arrow). Initially, we believed this encoded an ORF, but later found that it did not (discussed below). At this point the data suggested that a single locus was alternatively spliced, one transcript encoding exons 1–17, the other encoding exons 1–12 linked to exons 19–31.

We next amplified between primers in exons 9 and 21 and got slightly different results. Instead of an expected product of ca. 400 bp, we amplified a ladder of products, several which were larger than predicted from the 5′ RACE results. These products encoded the following exon combinations: the longest was exons 9–16∶18–21, the second was 9–15∶18–21, the middle was 9–14∶18–21, then 9–12∶19–21 (the product we had expected from the 5′ RACE results). In addition, there were several other smaller variants present. However, analysis of these products revealed that only a single product (9–14∶18–21) encoded an ORF. In every other combination, the downstream exon was out of frame and encoded multiple stop codons.

We extended these observations by making new primers in other exons and continued to amplify across this region using multiple combinations of sense primers encoded in exons 1–14, and antisense primers encoded in exons 18–31. In every case (12 different primer combinations), we could amplify a fragment, but most reactions amplified multiple products. An example in Figure 1C shows a typical amplification from exons 2–30, which amplified the expected 3.2 Kb product, as well as a number of smaller variants (arrows). Sequencing of these smaller products resulted in similar results to those described above, with multiple splice variants amplified. Only one of these transcripts encoded an ORF, which had exon 14 spliced to exon 18. At that point, it appeared that when we attempted to amplify the full-length cDNA, we also preferentially amplified rare splice variants because they were smaller than the full-length product, which we had reported previously [7].

In order to preferentially amplify the 14/18 linked transcript, we made both sense and antisense primers which spanned the 14/18 boundary, and amplified from there to the 5′ or 3′ end of the gene (both RACE and primers in exons 2 and 30), respectively [7]. In both cases we amplified a single product with no splice variants. Because we could amplify the whole gene (Figure 1C), and had a consistent 14/18 boundary, we concluded that there were two splice variants that encoded ORFs (the secreted form, exons 1–17, and the full-length form, encoding exons 1–14∶18–31), and that we were amplifying both productive and non-productive splice variants when we amplified across the 14/18 boundary. However, if we biased the PCR such that primers sat across the 14/18 boundaries, no splice variants were observed at all. In summary, we could always amplify across the 14/18 boundaries, but when we did we also amplified non-productive splice variants along with that encoding the transcript with the ORF (Figure 1C). However, if the downstream region included exon 18, no splice variants were observed (not shown). These experiments were repeated using different primer sets ca. 50 times with varying primer combinations and nest/hemi-nesting strategies, on animals from 5 populations on the east and west coast [7], [15].

Finally, we used a retroviral vector to express a construct consisting of exons 1–27 of the full-length transcript fused to GFP in 293T cells (Figure 1D). As shown, labeled protein from this construct was observed at the cell surface. This demonstrated that the fusion protein had folded correctly and transited through the secretory pathway to the cell surface. We have also expressed labeled versions of the secreted form in mammalian cells (not shown), and this was strong evidence that the *fuhc* was a single locus that expressed two alternatively spliced mRNAs, one encoding a secreted form, and one encoding a membrane bound form [7].

### The candidate *fuhc* locus likely encodes two tightly linked genes

**Figure 2:**
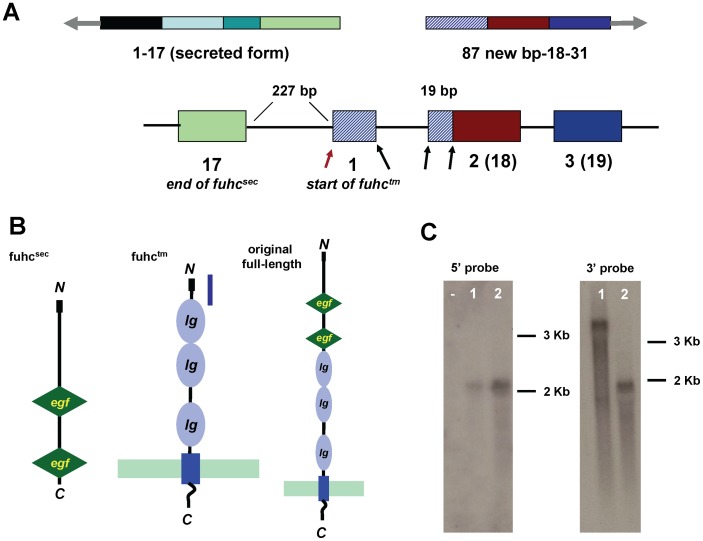
the candidate *fuhc* encodes two tightly linked genes. **A.** Schematic of the region between *fuhc* ^sec^ and *fuhc* ^tm^. The new 86 bp region is shown in hatched bars, and encodes a new start codon, and a portion of exon 2 of the tm form (exon number in parentheses are the original exon designation). Black arrows show correct intron/exon boundaries, the red arrow outlines the start of the first exon of *fuhc* ^tm^, which does not have the correct intron/exon boundary sequence. **B.** Illustration of the structure of *fuhc* ^sec^ (left) and *fuhc* ^tm^ (center) and original full-length protein (right) as predicted by ELM (17). The blue line on *fuhc* ^tm^ (center) represents the new coding sequence **C.** Northern blot of total RNA hybridized with probes specific for *fuhc* ^sec^ (left) and *fuhc* ^tm^ (rt). Left: lanes 1 and 2 are RNA isolated from two wild-type genotypes, – indicates no RNA. Right. Lane 1 is a positive control using RNA isolated from 293T cells expressing a construct encoding exons 1–27 of the original full-length *fuhc*/EGFP fusion protein (expressed in Figure 1D). Lane 2 is a separate genotype.

Over the last several years we have been creating a *B. schlosseri* transcriptome database using Sanger, 454 and Ilumina technology. When we analyzed the candidate *fuhc* from the assembled sequences, we found two sequences, one encoding the secreted form of the *fuhc*, and another encoding a shorter form encoding exons 18 to 31. We initially thought this was a logical output of the assembly program, as the shorter contiguous secreted form would dominate the assembly. However, we also noticed there was an extra 86 bp on the 5′ end of the 18–31 sequence that we had never encountered previously in any of our RACE or RT-PCR experiments. BLAST of this sequence to the genomic clone revealed that this extra 87 bp was encoded in the genomic clone in two regions (Figure 2A). The first 67 bp were encoded between exons 17 and 18, with the new sequence starting 227 bp downstream of the end of exon 17, while the remaining 19 bp were contiguous with the 5′ end of exon 18. In other words, exon 18 was 19 bp longer than we had reported previously (illustrated by hatched blue bars in Figure 2A). The new sequence is in frame with our original exons 18–31, and the initial 67bp encode a short 5′ UTR, a start Met, followed by a signal sequence, and the back half of the original c*fuhc* gene (Figure 2B). As described below, both genes are highly polymorphic and show signs of positive selection, and from herein will be referred to as *fuhc* ^sec^ and *fuhc* ^tm^.

The upstream region of the first exon of *fuhc* ^tm^ does not have any consensus exon/intron boundaries, but the 3′ region does. In addition, when we compared our original exon 18 with the longer exon 18, both showed the correct intron/exon boundary consensus sequences at the 5′ end of the exon, suggesting what we had originally identified and characterized as exon 18 in the full-length transcript was in fact a cryptic splice variant.

We followed up on these observations by doing northern blotting, 5′ RACE, RT-PCR and 5′ primer extension from original exon 18. Northern blotting (Figure 2C) was done on total RNA using probes from original exons 1–14 or 18–31. Both probes revealed the presence of only single transcripts of ca. 1.5 Kb, demonstrating that two main species existed (*fuhc* ^sec^ and *fuhc* ^tm^), and that our original conclusions were incorrect, as there is no detectable transcript of 3.2 Kb, which would have encoded the long transmembrane form originally reported [7].

We next did RT-PCR from primers in the new exon (exons 1–13 of *fuhc* ^tm^) and found that this transcript was expressed in all animals used in our crosses. Finally, we probed for the 5′ end of the transmembrane gene using both primer extension from a primer in exon 1, as we all as 5′ RACE from reverse primers in exons 21, 20, 19, and 18. As shown in Figure 1B (arrow), the reason we originally believed the *fuhc* encoded a splice variant expressing two genes was that 5′ RACE from exon 21 (now exon 4 of *fuhc* ^tm^, red arrow shows location of primer) amplified a 1.5 Kb product that extended to the first exon of *fuhc* ^sec^ , not the 350 bp product that we should have amplified (exon 4 to exon 1 of the *fuhc* ^tm^), given the fact that the *fuhc* ^tm^ is a single gene detected by northern blotting (Figure 2C). In other words, we amplified a 5′ RACE product that was seven times longer than the predominant transcript. When we took this RACE product and nested it with a primer in *fuhc* ^tm^ exon 2, we then amplified a much smaller product, which was the 5′ end of *fuhc* ^tm^. This new 5′ RACE product extended the original 67bp identified in the transcriptome database by 22bp, and all this new sequence was contiguous and encoded as additional 5′ UTR in the genome. The primer extension data gave equivalent sizes (not shown).

In summary, the c*fuhc* encodes two genes (Figure 2), the *fuhc* ^sec^, which is 17 exons in length, encodes a signal sequence and two EGF domains, as we had previously described (7; Figure 2B). The gene does appear to be secreted, as it has no GPI or other lipid attachment signatures. In addition, the signal sequence was functional in mammalian cells (Figure 1D), targeting the expressed protein to the plasma membrane. Following a 227 bp intergenic region, the *fuhc* ^tm^ is encoded in 15 exons, has a signal sequence, three predicted Ig domains, a transmembrane domain in exon 11, and an intracellular tail with no known signaling domains (Figure 2B).

The end of *fuhc* ^sec^ is 227 bp in front of the beginning of *fuhc* ^tm^. This small intergenic region is nearly featureless and has no predicted promoter elements (Figure 2A; sequence found in Figure S1).

### Expression of the secreted and membrane-bound forms

We localized expression of the two genes usin g both qPCR of isolated tissues and whole mount in situ hybridization. Table 1 shows typical qPCR results from blood and ampullae of a single genotype, using ddCT versus expression of the housekeeping gene ef1α. The *fuhc* ^sec^ is expressed at a significantly higher level than *fuhc* ^tm^ in both tissues. In addition, expression of both transcripts in the blood is slightly higher than that in the ampullae. However, the ampullae are part of the vasculature and are always contaminated with blood, and this is likely the source of the large variability in the data.

**Table 1:** qPCR expression levels of the *fuhc* in ampullae and blood.

Tissue	ddCt *fuhc* ^sec^	ddCt *fuhc* ^tm^
Ampullae	120+/−56	1.8+/−0.77
Blood	129+/−49	5+/−1.8

Expression was measured using a ddCt methodology with the housekeeping gene ef1α as the standard. Values and SEM are shown for 5 biological replicates from a single genotype.

**Figure 3:**
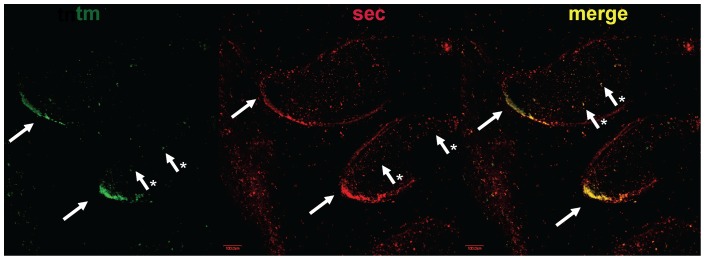
Localization of *fuhc* ^sec^ and *fuhc* ^tm^ using whole mount double-labeled in situ hybridization. Probes specific for *fuhc* ^sec^ and *fuhc* ^tm^ were hybridized to whole mount preparations as described in the methods, and results are shown for *fuhc* ^tm^ (green) and *fuhc* ^sec^ (red) as well as the merged image. Both genes are expressed in the epithelium of the ampullae as well as a subset of blood cells. Note that while detection of *fuhc* ^sec^ is repeatable, detection of *fuhc* ^tm^ was variable, in that we saw either this staining pattern, or no detection of *fuhc* ^tm^ mRNA at all. This is most likely due to low levels of expression (Table 1).

Double-labeled *in situ* hybridization (ISH) was used to locate expression of both secreted and membrane bound forms of the *fuhc* (Figure 3). In adults, we found that the secreted protein was expressed in both the ampullae as well as a subset of blood cells. This is equivalent to our previous results, which had used a probe in the same region (exons 1–14 of the secreted form). The membrane-bound mRNA was also expressed in the tips of the ampullae as well as blood cells, although at a much lower level than that of the secreted form (Figure 3). This difference may be due to the low level of expression of *fuhc* ^tm^, which can be difficult to detect using these methods (discussed below)- in multiple experiments, we saw either expression in the ampullae and low levels in blood (shown in Figure 3) or no expression at all in either tissue. This is most likely due to the very low expression of the *fuhc* ^tm^ mRNA (Table 1).

### Linkage of the two transcripts

Given the northern blot results, it is clear that the candidate *fuhc* region encodes two transcripts, not a single, alternatively spliced transcript we had previously reported. However, it is unclear how we can amplify products that encode exons from both genes if they are independent transcription units. Moreover, the 227bp intergenic region that links the end of secreted protein to the beginning of the transmembrane protein is nearly featureless, and has no significant promoter elements. It is also very small, as most intergenic regions between known transcripts in the sequenced region of the *fuhc* (ca. 1.2 Mbp) average about 3 Kb [6].

**Figure 4:**
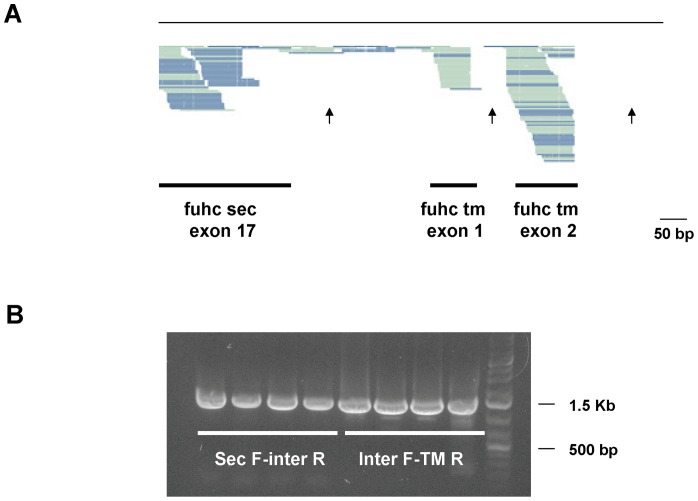
A stable transcript encoding both *fuhc* sec and *fuhc* tm can be detected by mRNA-seq and RT-PCR. *Top* Alignment of Illumina mRNA-seq reads from a single genotype to the *fuhc* genomic region spanning from *fuhc* ^sec^ exon 17 to the intron following *fuhc* ^tm^ exon 2. Multiple sequences span the 227bp region between the genes (arrow in center), however, there are no aligned sequences in introns between *fuhc* ^tm^ exons 1 and 2, or 2 and 3 (right two arrows). *Bottom* RT-PCR on cDNA isolated from 4 wild-type individuals. The right 4 lanes are from the exon 2 of *fuhc* ^sec^ to the intergenic region, the left 4 lanes are from the intergenic region to the exon 13 of *fuhc* ^tm^. Only single products are amplified using these primers.

One explanation for our results would be if the *fuhc* was an operon, a two gene transcript which is trans-spliced into two independent messages [18]–[19]. Operons have been identified in tunicates and trans-splicing has been described previously in *B. schlosseri* [20]–[22]. To assess if transcripts linking the two genes existed, we initially aligned mRNA-seq reads from different genotypes to the genomic sequence. A representative alignment from a single genotype is shown in Figure 4A, and reveals that there were overlapping sequences that linked the last exon of the secreted form to the first exon of the membrane-bound form, although reads spanning the intergenic region are found at a much lower frequency than those which align to other exons. In contrast, introns can be seen in this alignment (arrows) that had no coverage, demonstrating that mRNA-seq reads aligning to the intergenic region were not due to genomic DNA contamination (Figure 4A).

We followed up on this result by amplifying from primers in the intergenic sequence to the 5′ end of the secreted form and the 3′ end of the membrane-bound gene (Figure 4B). In both cases, we identified single transcripts encoding the correctly spliced cDNA with no missing exons, and no smaller amplicons present. In summary, if the intergenic region was present, we amplified a single product. However, if amplification was done using primers that spanned the same region, the result was a ladder of alternatively spliced products (compare Figure 4B to Figure 1C). As both the mRNA-seq libraries and cDNA preparations used in the alignments and PCR experiments were made using CAP-trapped, polyA isolated mRNA, this suggests the presence of a single, capped, polyadenylated transcript the encodes the correctly spliced exons 1–17 of the *fuhc* ^sec^, contiguous with the 227bp intergenic region and the correctly spliced exons 1–15 of *fuhc* ^tm^.

If the *fuhc* was an operon, and this long amplicon represents a stable intermediate, we would expect that the second gene would be trans-spliced, and should contain a Splice Leader (SL) sequence [18]–[22]. However, both 5′ RACE and primer extension results were equivalent, suggesting that the second transcript does not contain a SL sequence at the 5′ terminus. In summary, there is low-level expression of a single transcript that unites the two spliced messages via the intergenic region. While it is possible that this indicates a unique trans-splicing event that does not include the addition of a SL sequence, it also could be due to a read-through of RNA polymerase between the two genes that occurs occasionally but is not biologically significant (discussed below).

### *fuhc* polymorphisms and fusibility

We recently found that both genes are highly polymorphic and show evidence of positive selection, suggesting they are both involved in histocompatibility, and this is consistent with their expression patterns (Figure 3) [15], [28]. Next we tested which of the two genes determined outcome, or if fusibility is due to a haplotype of the two genes together. In previous experiments we had focused on the 5′ end of the gene (now *fuhc* ^sec^), as it was the most polymorphic region of the gene [7]. We initially re-tested our most rigorous example, a fusion occurring between individuals from different geographic locations (Table 2; Figure S1). An F1 between an individual collected from Half Moon Bay CA and a colony from Monterey CA (HM9A ×1225 F1) fused with each of two non-sibling individuals from Santa Cruz, CA (SC 32e and SC 27f), however the two Santa Cruz individuals rejected each other. We amplified both *fuhc* ^sec^ and *fuhc* ^tm^ from all three genotypes, and the alleles matched outcomes exactly at nucleotide sequence. Each individual was heterozygous, and the two Santa Cruz individuals shared one allele with the Half Moon Bay individual, but none between them.

**Table 2:** *fuhc* ^sec^ and *fuhc* ^tm^ alleles correlate with fusion/rejection outcomes in wild-type individuals.

Pairing	Fusibility Assay Outcome	*fuhc* ^sec^ alleles shared	*fuhc* ^tm^ alleles shared
(HM9aY x 1225 F1) vs SC32e	Fusion	1	1
(HM9aY x 1225 F1) vs SC 27f	Fusion	1	1
SC 32f vs. SC27f	Rejection	0	0
SB 1 vs SB 2	Rejection	0	0
SB 1 vs SB 3	Rejection	0	0
SB 1 vs SB 4	Rejection	0	0
SB 1 vs SB 6	Fusion	1	1
SB 2 vs SB 3	Fusion	1	1
SB 2 vs SB 4	Fusion	1	1
SB 2 vs SB 6	Rejection	0	0
SB 3 vs SB 4	Fusion	1	1
SB 3 vs SB 6	Rejection	0	0
SB 4 vs SB 6	Rejection	0	0

Wild-type individuals were reared in the lab, grown and subcloned into multiple naïve pieces. One group of subclones from each genotype was paired and fusion/rejection outcomes visually assessed. Naïve subclones of the same individuals were isolated for one week to ensure they were not pregnant, then sacrificed for RNA isolation and subsequent cloning and sequencing of the both alleles. cDNA and protein alleles are shown in Figure S1.

We followed this by studying lab-reared, wild-type genotypes that were progeny of colonies collected in Santa Barbara, CA. Following a round-robin experiment using multiple individuals, we picked a subset of five genotypes which showed nearly equivalent amounts of fusion or rejection when tested against each other (4 fusions and 6 rejections). We cloned and sequenced the majority of the ectodomain of both genes from each individual. All individuals were heterozygotes at both alleles, and fusing colonies shared an allele at both *fuhc* ^tm^ and *fuhc* ^sec^, while rejecting colonies did not share alleles at either locus. Thus far, polymorphisms on both *fuhc* ^sec^ and *fuhc* ^tm^ correlate with fusion/rejection outcomes, and this is true both within and between populations (Table 2; cDNA and protein sequences found in Figure S1).

While these results confirm the correlation between polymorphisms on the *fuhc* locus and histocompatibility, they do not discriminate between which of the two genes determines outcome. We have not yet found a pair of wild-type *fuhc* haplotypes in which *fuhc* ^sec^ alleles matched, but the *fuhc* ^tm^ alleles were disparate, or the reverse case. Given that the two genes are only 227bp away from each other, it will not be easy to find these genotypes. In summary, at this point we do not know if one gene or the other is the allodeterminant, or if both are together. However, as described below, we recently re-analyzed some of our original cross data, which sheds some light on this question.

### Original cross genetics

The final unresolved issue from our original positional cloning project regarded one of our inbred *fuhc* homozygous strains, called AA1023, which was a parental genotype in the main F2 mapping cross (6, 23), and may lend insight into polymorphisms and fusibility.

During our initial analysis of the candidate gene, we amplified a small intron between exons 20 and 21 of the c*fuhc*- now called exons 4 and 5 of the *fuhc* ^tm^ – called STS1 [6], [7]. This intron was polymorphic and had several SNPs encoded within restriction endonuclease sites, which allowed us to discriminate between *fuhc* alleles in multiple crosses using a PCR-RFLP strategy, and STS1 polymorphisms segregated absolutely with allorecognition outcomes in multiple crosses [6], [7].

Surprisingly, we found that our parental *fuhc* homozygote AA1023 was heterozygous at STS1, encoding two variants (called A1 and A2). Both A1 and A2 intron sequences were unique and different than the B and Y sequences. However, amplification of cDNA from the same genotype only revealed a single cDNA sequence using multiple primer sets [7] and done independently by several people over the last 8 years. Conversely, the *fuhc* genes are so polymorphic that this result in and of itself is not conclusive; primers could bind differentially, leading to a PCR amplification bias. Since we had gotten the expected result (a single cDNA sequence from a homozygote), we next focused our efforts on characterizing the candidate gene, and did not follow up on this observation at the time.

However, given that the present study is due to strange PCR results, we re-analyzed these animals, as the STS1 polymorphism could indicate that the transmembrane gene had changed between the A1 and A2 genotypes during derivation and maintenance of our lab-reared strains, and we had missed it. Thus, we amplified both *fuhc* ^sec^ and *fuhc* ^tm^ from cross progeny containing the A1 genotype but not A2, which we had not done previously. We found that our homozygote AA1023 parental strain was not a homozygote at all, it was actually heterozygous, and encoded two very distinct alleles at both *fuhc* ^sec^ and *fuhc* ^tm^. While surprising, due to the fact that we used the same AA1023 genotype as the tester strain to genotype our F2 individuals, and in addition used a bulk segregant approach to screen for polymorphic markers linked to fusibility outcomes for genetic mapping, this did not affect any aspect of positional cloning of the locus (discussed below).

We followed up on this observation by testing different individuals derived over a 10 year period which were genotyped as AA homozygotes (via histocompatibility assays to other individuals) using STS1. We found that our original cross progeny were A2 homozygotes, and that the STS1 was not introduced into our AA lines until ca. the 4^th^ generation of inbreeding within the lab (Table 3) [6], [23]. Following introduction of this contaminant allele, it was inherited in a normal Mendelian fashion, with subsequent generations containing both homozygotes and heterozygotes of A1 and A2, and AA1023 was a heterozygote we used for one of our mapping crosses.

**Table 3:** Segregation of the A1 and A2 intron alleles.

Name	Genotype
P11-1r	A2 homozygote
Yw746	A2 homozygote
Yw1153	A2 homozygote
Yw1352	A1/A2 heterozygote
Yw1023	A1/A2 heterozygote
Yw1328	A1 homozygote

Shown are individuals from our mariculture pedigree which were phenotyped as a *fuhcA* homozygote by fusibility assays, and their corresponding genotype by the STS1 PCR-RFLP marker, which detects polymorphisms in an intron between exons 4 and 5 of the transmembrane gene. Yw1023 was a parental line in our main mapping cross. Pedigree placements of these genotypes in these *fuhc* inbred lines can be found in [23].

Since both the secreted and transmembrane proteins were different in AA1023, this did not answer the original question of which gene controlled outcome. However, analysis of the A1 sequence may in fact be providing some insight into the role of the two proteins. During our genetic mapping studies [6], [7], [23], we scored F2 genotypes for their fusibility type by placing them in contact with AA1023, and fusion or rejection was visually assessed. In our main mapping cross (n = 83) only a single animal was ambiguously scored. That individual was F2#20, which was later genotyped as a *fuhc*BB homozygote, the only B homozygote in the population [6], [24]. In multiple naïve pairings of this individual and AA1023 n = 5), no phenotypic rejection response occurred. However, fusion did not occur either. It was noted that at the initiation of contact the ampullae from each individual penetrated deep into the other individual (which is never seen in a rejection response), but the fusion site and subsequent blood transfer was never observed. These individuals should have rejected, and this repeatable phenotype was genotype and pairing specific: AA1023 rejected all YY and BY F2 individuals robustly.

It had been previously observed that severity of rejection maps to the *fuhc* locus, suggesting that the strength of the rejection response could be due to the differences between alleles [25]. In addition, we have recently shown that histocompatibility in *B. schlosseri* works by a missing-self mechanism [16], whereby rejection is initiated by an independent pathway and occurs unless a self-allele is recognized on the other individual. Upon recognition of self, a fusion pathway is initiated that overrides the rejection response and stimulates vascular remodeling, eventually resulting in parabiosis [9]. Given the integration between these two pathways, we would predict that pairings with individuals carrying closely related alleles would partially recognize each other, moderately stimulating the fusion pathway, in turn lowering the severity of rejection. However, at the time this was not a satisfactory explanation, as the A (now A2), B and Y alleles for both secreted and tm forms are equally divergent (Table 4). When we compared the new A1 alleles to B and Y, we found that the *fuhc* ^tm^ were equally divergent among all three alleles. However, analysis of *fuhc* ^sec^ gave a different result: while A1, A2 and Y are all divergent, A1 and B differed by only 6 amino acids and four of those changes were with amino acids with equivalent charges (Table 4). The rejection phenotype between AA1023 and F2#20 was repeatable in multiple naïve pairings of these genotypes (n = 5), and of all the pairings we have done, these are the most closely related alleles at the *fuhc* ^sec^. Together, this suggests that it may be the secreted form that either determines or dominates the response.

**Table 4:** Amino Acid Changes in Cross Alleles.

Alleles	Total Differences	Identities	Non-identity
**Transmembrane**			
A1 vs B	27	14	13
A2 vs B	27	13	14
A1 vs Y	9	4	5
A2 vs Y	10	4	6
B vs Y	18	10	8
**Secreted**			
A1 vs B	***6***	***4***	***2***
A2 vs B	22	12	10
A1 vs Y	20	10	10
A2 vs Y	30	13	17
B vs Y	24	11	13

The number of amino acid changes between alleles in both transmembrane and secreted forms of the *fuhc* are shown for the main F2 mapping cross [6]. For the transmembrane form, the region analyzed is from exon 2–11, encompassing the entire ectodomain. For the secreted gene, the analysis included exons 4–14. Exons 15–17 are not polymorphic.

## Discussion

We have found that the candidate *fuhc* locus is much more complicated than previously described, and contains two transcripts, one secreted and one membrane bound, that are linked by a 227bp intergenic region, rather than a single, alternatively spliced gene, as we had previously reported [7]. Despite this newly discovered complexity, this still appears to be the histocompatibility locus: both genes are expressed in the ampullae and a subset of blood cells, and polymorphisms predict histocompatibility outcomes by themselves. To add to this complexity, we have also found another polymorphic gene encoded ca. 8 Kb away from the end of the *fuhc* ^tm^ locus that encodes a hsp40 family member, which is currently being analyzed [34]. Similar to the *fuhc* proteins, it is expressed in the ampullae and shows evidence of positive selection, all consistent with a potential role in histocompatibility.

The finding that there are two genes in the candidate *fuhc* has several implications and is quite confusing. First, although both genes are among the most polymorphic proteins ever described, we have found that the secreted gene is much more diverse, and has many more residues that show evidence of positive selection versus the membrane bound gene [15]. In the one ambiguous fusion/rejection outcome we saw in our mapping crosses, we found that the secreted forms were much more closely related than the transmembrane forms, suggesting the secreted protein dominates the allorecognition response, and this observation is consistent with results from an independent study (discussed below) [26]. However, it is difficult to understand how a secreted protein could confer self/non-self recognition information between juxtaposed ampullae, as once released from one individual the source could no longer be known. It may be that the secreted form is not the ligand, and that the *fuhc* ^tm^ is, and we have not yet found an example where a match at the secreted gene and a mismatch at the membrane-bound gene causes a rejection. However, this is inconsistent with data presented here, as well as the molecular evolutionary studies, and we would expect the most polymorphic gene to be the ligand. Finally, while there are no clear GPI or other lipid attachment signatures, it is also possible that *fuhc* ^sec^ may be held in a complex on the cell surface with other proteins.

Alternatively, given that they are separate genes, it may be that the *fuhc* ^sec^ and *fuhc* ^tm^ work via a lock and key interaction. However, this does not make complete sense either, as the source of a secreted ligand would still be unknown to the receptor that bound it, regardless of what that receptor is. Moreover the idea that tens to hundreds of specificities have evolved via a lock and key mechanism seems far-fetched from a structural viewpoint. This would imply that a ligand would mutate, followed by a receptor having a complementary mutation such that it binds only its ligand instead of multiple other, closely related ligands, and moreover that this highly-specific co-evolution occurred hundreds of times, randomly. Finally, given the single allele match rules of histocompatibility, it cannot be that the interaction of a haplotype of *fuhc* ^sec^ and *fuhc* ^tm^ block fusion.

### Linkage of the two genes

Northern blotting and mRNA-seq results clearly demonstrate that there are two major transcripts. However, we can also detect linkage both across the intergenic region as well as splice variants that link the two genes by both PCR and mRNA-seq. While it is clear that these linked transcripts are rare, the question is why they exist at all, and are stable enough that we can amplify multiple variants. There are two major hypotheses. First is that the two genes are in fact part of an operon, and transcribed together, then separated. Operons and trans-splicing have been described in tunicates previously, and the intergenic region is devoid of any predicted promoter elements [18]–[22]. However, as discussed above, if this is true we would expect to see the *fuhc* ^tm^ to have a splice leader (SL) sequence, and this is not observed. It could be that we are seeing a novel regulatory event, essentially trans-splicing without SL sequences. However, proving this would require multiple tools not yet available in this system, specifically transgenic lines, and in many ways it seems unlikely, although it cannot yet be dismissed either.

The second hypothesis is that the single linked transcript is due to a low level of read through of the RNA polymerase between the short intergenic region between the two genes, and is not part of any regulatory process or biologically significant. If that was the case, it may not be surprising if exons between the two genes were occasionally spliced together. We favor this hypothesis because it explains nearly all of our observations to date. First, we had previously noted that there were non-productive splice variants detected whenever we amplified across what we believed was the alternatively spliced exons (the original exon 14∶18 splice junction). These would have been preferentially amplified as they were shorter than the expected full-length product. Second, the beginning of this original exon18 is not even correct, and is actually 19 bp within what we now know to be the correct exon sequence (Figure 2A). Non-specific splicing could explain this observation, as both the old and new 5′ start site of that exon have canonical intron/exon 5′ splice sequences.

More importantly, we had never identified any transcripts that included the first exon of the *fuhc* ^tm^ or the intergenic region in any previous experiment. This is also consistent with random splice events, because if splicing occurred between the two transcripts, these regions would never be included. The first exon of the *fuhc* ^tm^ does not have canonical intron/exon boundaries at the 5′ end of the exon, thus any splicing that occurred between the two genes would delete this region, as the first 5′ splice site available would be downstream of the first exon (Figure 2A). In support of this conclusion, if we PCR from the intergenic region to the end of either gene, we amplify a correctly spliced transcript (Figure 4B). In other words, if the intergenic region is present on a transcript, the two genes are linked and introns correctly processed. The presence of this linked, correctly processed transcript is supported by deep sequencing of poly A, cap trapped RNA, which have sequences that cover the intergenic region (Figure 4A). In summary, multiple stable transcripts that contain full or partial *fuhc* ^sec^ splice to exons in *fuhc* ^tm^ can be identified by RT-PCR, RACE and independently by deep sequencing, although these are clearly rare and not detected using less sensitive methods, such as northern blots (Figure 2C).

In retrospect, the most difficult result to understand is how we would RACE one of these rare products preferentially. The original conclusion that the *fuhc* was a single, alternatively spliced gene was based on 5′ RACE from exons in the *fuhc* ^tm^ gene that extended into the *fuhc* ^sec^ gene (Figure 1B, arrow). In these experiments, primers from the *fuhc* ^tm^ amplified fragments that included the 5′ end of *fuhc* ^sec^ and were ca. 1.5Kb in length, when the predominant product (the front end of *fuhc* ^tm^) should have been ca. 350 bp in length. This is still difficult to understand. If rare transcripts with incorrectly spliced exons are present, it would not be unexpected to detect them using RT-PCR when amplifying across that region (Figure 1C). However, amplifying a rare long product using RACE when the major competing product is 7X smaller and clearly orders of magnitude more abundant (Figure 1B; arrow) is not easy to explain. This may indicate a secondary structure on the 5′ end of the *fuhc* ^tm^ transcript which inhibited the template switching required by the SMART 5′ RACE procedure, or elongation by Taq. In support of the latter, we only got the 5′ RACE of the *fuhc* ^tm^ to work when we used a nested PCR strategy and the second primer was within 120 bp of the transcription start site. While this observation could also suggest the presence of a non-canonical 5′ CAP, we believe the *fuhc* ^tm^ has a normal capping structure, as it was first identified in 5′ Sanger ESTs made from cap-trapped, poly A mRNA, and cap-trapping often will not bind to non-canonical 5′ caps [33].

The last strange observation is that linkage of the two transcripts was only seen in 5′ RACE. All 3′ RACE products from exons in *fuhc* ^sec^ ended at the end of the *fuhc* ^sec^ transcript, and we never identified an amplicon that crossed into *fuhc* ^tm^, although we crossed the exact same region going the other way. However, in context of our original two splice transcript model, this always made sense- a 3′ RACE from exon 9 should preferentially amplify the secreted form- it is 1.5Kb smaller than what we believed was the membrane-bound splice variant. So the 5′ RACE results suggest some other characteristic of the 5′ region of the *fuhc* ^tm^ transcript affected our results.

In summary, it is clear from northern blotting that the *fuhc* is in fact two genes. While there may be unique trans-splicing event that generates two transcripts, it seems more likely that we were misled by 5′ RACE results due to a biologically insignificant read-through event. Conversely, it should be noted that exons 1–27 of the originally described, full-length gene can be expressed in mammalian cells and folds correctly (Figure 1D). This seems remarkable given that it is essentially a random fusion of two genes, with an incomplete exon in the middle of the mRNA (Figure 2A). It may be that this form is expressed at a very low level, and not detected by Northern blotting, as it is clear that a repertoire of stable, transcripts exist that encode the originally described full-length and smaller parts of both genes (Figure 1B,C). Finally, the most polymorphic region of the locus encodes a predicted secreted protein, and this negates our original ideas on the structural basis of recognition that underlies this highly polymorphic histocompatibility system. Unfortunately, we do not yet have the tools to look at single tissues at high resolution, nor formally test the possibility that we are observing some novel regulatory event. The only thing that is clear at this point is that no explanation is completely satisfying.

### *fuhc* ^sec^ and *fuhc* ^tm^ polymorphisms and histocompatibility

To date, we have not found a case where the *fuhc* polymorphisms do not correctly predict histocompatibility outcomes. Moreover, both genes encode proteins that are extraordinarily polymorphic, and clearly evolving under natural selection for diversification [15], [28]. However, at this point we do not know if both genes play a role in outcome (a haplotype of *fuhc* ^sec^ and *fuhc* ^tm^), or one or the other protein dominates. This will require finding genotypes where one locus matches and the other does not, and will not be trivial given their close linkage. In addition, we are continuing to test both genes functionally using siRNA-mediated knockdown as well as direct binding assays and other *in vitro* techniques, although due to multiple technical hurdles, these experiments have not progressed as quickly as we would like.

A recently published report concluded that *fuhc* polymorphisms did not predict histocompatibility outcomes [26], based on results from four experiments equivalent to those performed here: correlating fusion/rejection outcome to the *fuhc* alleles expressed by the interacting genotypes. However, the experimental design and conclusions were not robust.

In three of the four experiments, only a small region of the *fuhc* ^tm^, encoding either 45 or 167 amino acids (< 5% and <20% of the originally described ectodomain, respectively) from the interacting genotypes was analyzed. It was claimed that there were 13 discrepancies between histocompatibility outcome and alleles at these regions. In 12 of 13 discrepancies, individuals in a pairing rejected even though they shared an allele, and it was concluded that the fuhc was not predicting fusion/rejection outcomes [26]. Actually, no conclusions can be made from discrepancies involving rejection responses as described [26]: it is not possible to discriminate between alleles using markers that leave out 80–95% of the sequence encoding the ectodomain [15].

The final discrepancy was a single pairing where the 167 residue region was polymorphic between individuals, and it was shown that two individuals that should have rejected had fused. However, in this case, one of the two individuals was genotyped as being homozygous. In our experience, assigning an individual as a homozygote using a single PCR-based genotyping experiment, as was done in that study [26], is not definitive. First, as shown here, we missed the heterozygosity of AA1023 using multiple primer sets and over 100 independent subclones. Second, in a recent study, we analyzed a minimum of 16 sequences/individual, and even then did not always detect the second allele until a replicate experiment had been done, and that usually required the use of different primers [15]. *fuhc* genotyping in the conflicting report was based on a single experiment in which only 8 independent subclones/individual of a single amplicon were analyzed [26], and there may have been a second allele that was not found. Along those lines, it is important to note that in no case did two heterozygous individuals reject when they should have fused [26]. In addition, with the exception of two cases described below, it should also be noted that there are no examples in which the regions analyzed were polymorphic between individuals and the two fused when they should have rejected. If *fuhc* polymorphisms truly had no affect on fusion and rejection outcomes, equal amounts of both cases should have been identified.

In the last of the four experiments nearly the entire secreted region was sequenced (a region encoding 455 amino acids) and correlated to fusion/rejection outcome. In two pairings it was stated that the two individuals did not share *fuhc* ^sec^ alleles but had fused. Remarkably, analysis of the alleles revealed that in both cases the individuals in each pair expressed *fuhc* ^sec^ alleles that differed by a single amino acid over this 455-residue region [26].

We recently analyzed a large database of *fuhc* ^sec^ alleles, and found that the average difference between any two alleles was over 20 amino acids, and the closest pair of alleles we have identified had 6 differences (Table 4), thus finding one with a single change was fortunate and may be insightful from a functional standpoint. However, concluding that this is evidence for *fuhc* ^sec^ not playing a role in allorecognition is not credible. It would not be surprising if a single amino acid change between two alleles does not confer enough changes in structure of the protein to be detected as a new specificity type. Even T-cells cannot detect every amino acid change in their MHC ligand(s), particularly if the substitution was outside the peptide-binding region, and *B. schlosseri* uses an innate effector system to discriminate between *fuhc* alleles [2]. Given that we have no idea how an innate recognition system can discriminate between hundreds of *fuhc* alleles, one of the most intriguing structural questions in this field revolves around the mechanisms of recognition and evolution of specificity. Molecular evolutionary studies of both receptors and ligands provide insight into these questions, as characterization of the amount and distribution of polymorphisms between alleles, as well as which residues show evidence of positive selection, reveal regions of the proteins which must change to provide changes in phenotype, i.e., specificity [15], [27]. So if a single residue change between two alleles of *fuhc* ^sec^ does not change the fusibility outcome, the location and type of substitution is actually an insightful finding, and completely consistent with our results (Table 4) indicating the role of *fuhc* ^sec^ in outcome. It is unfortunate that we do not know the *fuhc* ^tm^ alleles in the two pairings from that study [26].

In summary, we have not found a case where *fuhc* ^sec^ and *fuhc* ^tm^ do not predict outcome, even in genotypes isolated from different locations (Table 2). Conclusions from a previous study which suggested that polymorphisms do not correlate with outcome [26] were based on experiments that did not test the original hypothesis that polymorphisms in the originally described ectodomain predicted outcome, and only small fragments of the genes were examined. Thus any discrepancy that involved a rejection response (13 of the 16 reported) is not conclusive. In the cases of the three inappropriate fusion events, in one case one of the individuals was homozygous, which could be an artifact of the genotyping methodology, and in the other two cases, the fuhc^sec^ alleles differed by a single amino acid out of the 455 residue region analyzed. If true, this latter result is significant and provides strong corroborating and independently derived evidence of the correlation of polymorphisms in *fuhc* ^sec^ and allorecognition outcomes [26].

The potential role of *fuhc* ^sec^ in the response was further substantiated by our surprising finding that one of our strains was not correctly genotyped, and that the only ambiguous scoring of an allorecognition outcome between two individuals correlates to the amount of differences in *fuhc* ^sec^ , but not *fuhc* ^tm^ (Table 4). Fortunately, our genetic mapping strategy was not affected by the presence of this contaminant allele, as we used bulk segregant approach to screen through thousands of molecular genetic markers to identify those that segregated absolutely with what we called the A allele in all generations of the cross [6], [23]. In turn, the presence of the A allele was scored in cross progeny via fusion to AA1023, thus the presence of the other allele was not detected using this assay, as all F1 and most F2 individuals fused with the heterozygous parent. In summary, our bulk segregant approach was actually screening for polymorphisms in the A1 and A2 alleles that were not in the B or Y alleles, and since the parental strain was both a heterozygote and also the tester strain, segregation of the ‘A’ allele appeared normal.

Prior to our mapping efforts, all *fuhc* genotyping was done via histocompatibility reactions using individuals derived from our individual founder colonies [23], and somewhere during derivation and maintenance of the A containing lines a new allele must have been introduced that was not detected. As shown in Table 3, the new allele does not appear until the 4^th^ generation of inbreeding in the lab, and from that point on each of the alleles segregates normally, producing both homozygotes and heterozygotes. Since all colonies were tested to each other for genotyping, the contaminant allele would only have been detected if an A1 and A2 homozygote had been picked for a pairing. So far, we have only detected a single A1 homozygote in those individuals, so this is not unexpected.

These results are not surprising given the biology of allorecognition in *B. schlosseri*. Following fusion of compatible individuals, germline progenitors can transplant between individuals and contribute to germline development in the parabiosed partner [29]. Further, we have found that this can occur between newly metamorphosed juveniles and adults, which could be easily missed during normal rearing. Juveniles are tiny, can land on top of an adult, and following fusion can be resorbed by the adult colony within a few days- but still contribute to the germline within 14 days [30].

Importantly, once a contaminant germline was introduced, it would be strongly selected for, as adult *fuhc* homozygotes are very difficult to produce. In the lab we have previously shown an unusual selection on the *fuhc* locus that strongly favors survival of heterozygous individuals to adulthood. In multiple crosses, *fuhc* homozygotes were born at normal Mendelian ratios, but die non-randomly prior to sexual maturity, the reason this occurs is not understood [31]. Moreover, this was not the first time an individual genotyped as a *fuhc* homozygote was later determined to be heterozygous. One of our original F2 cross parental genotypes was thought to be a BB homozygote, and only months later did we discover that it was a BY heterozygote [23]. Since all individuals used in the crosses were naïve, (i.e., they were never involved in a histocompatibility reaction), the only viable explanation is that germline transfer via juvenile/adult fusion contaminated our lines. It should be noted that this chimerism is very easy to replicate experimentally [30], and we believe germline transplant between juveniles and adults is likely the core interaction that controls evolution of histocompatibility in this species [32].

Ironically, this contamination provided some insight into the structural question of genotype versus phenotype for *fuhc* alleles. We are currently doing a large-scale experiment to correlate the severity of rejection to the amount and distribution of polymorphisms on both the *fuhc* genes from wild-type colonies, with the working hypothesis that two individuals with closely related alleles would show a weaker rejection versus those with divergent alleles. Out of pairs of animals we have picked randomly and analyzed thus far, we have not yet identified two *fuhc* ^sec^ alleles that are as closely related as A1 and B. It should also be noted that this result demonstrates an expected dose-dependence to the interaction between alleles: both YY and BY genotypes rejected the A1/A2 individual robustly, but the BB genotype did not. Furthermore, an independent study suggested that *fuhc* ^sec^ alleles which differed by a single residue were not detected as being different [26], and is entirely consistent with this result.

To conclude, we have found that the candidate *fuhc* gene is not a single, alternatively spliced gene as previously described. Rather, it is two genes linked together by a short intergenic region. While we can detect a single transcript that contains the processed forms of both genes using two independent techniques, northern blotting demonstrates that the vast majority mRNAs of each are a single species. The detection of single transcripts linking the two genes might suggest that the two genes are transcribed as an operon which is later trans-spliced into two mRNAs; however we cannot detect any other characteristics that would support this conclusion, such as a splice leader sequence on either message. Moreover, a rarely occurring read-through by the RNA polymerase coupled to splicing events which link the two genes together seems to be the simplest hypothesis that can explain all our results, both here and in our initial characterization of the gene: however, simple does not mean true, and we do not yet have the tools to discriminate between the two hypotheses. Nevertheless, both genes are highly polymorphic, show signs of positive selection, are expressed in tissues involved in histocompatibility, and polymorphisms correlate with allorecognition outcome. Understanding how both genes function in histocompatibility in concert with other genes is our current focus.

## Supporting Information

Figure S1 **A.** *fuhc* ^sec^ and *fuhc* ^tm^ cDNA and translated protein alleles from individuals used in Table 2 are shown. Primer sequences are included and described in the Methods. There is no concordance between the numbering of *fuhc* ^sec^ and *fuhc* ^tm^ alleles; i.e., no haplotype information is known. **B.** Sequence of the intergenic region between *fuhc* ^sec^ and *fuhc* ^tm^.
(DOC)
