# Supplementary material for: The Candidate Histocompatibility Locus of a Basal Chordate Encodes Two Highly Polymorphic Proteins
Source: PLoS One. 2013 Jun 24;8(6):e65980. doi: 10.1371/journal.pone.0065980 (PMC3691228; doi:10.1371/journal.pone.0065980)
Supplement: Figure S1 — A. fuhc sec and fuhc tm cDNA and translated protein alleles from individuals used in Table 2 are shown. Primer sequences are included and described in the Methods. There is no concordance between the numbering of fuhc sec and fuhc tm alleles; i.e., no haplotype information is known. B. Sequence of the intergenic region between fuhc sec and fuhc tm. (DOC) [file pone.0065980.s001.doc]

**Figure S1**

**A. cDNA and protein alleles of fuhc sequences from Table 2.**

**fuhc secreted cDNA sequences**

>HM9AYx1225F1_sec_allele_1

GAAATGTTGCTGAAAATATTCTGTCTTCCTTGAGCAATGACGACATTACTGCTATCCAGACCGGCTACGATTCTCCGTT

GGTATTTTCTGGAATATTATTCGCCGGATCGCAAATCGTCTATTTGGAAAACCTGGTGCAAAGGCTGAAATCTAACGTT

CCCTTATTATCTCCATCGGACTCTGGCGAATTTGAGGGATCAGAGTATGCTGTCAGGACAGGATGGCCTTATATTCATA

TCGCTTACTTCAATAATTCGGACCGATCGCGCGAGAGTGTTTCAGCTGGTATCAAAACTACCCCAAGTGGCCTTACCTT

TAGCACACCAATCAACACTACAGGAACCATTGATTCTTGTACCTACTCAGCTTATACCGTGCTGTCACTGAAGGAAGAC

GAAACAAGTTCAGTGGAATTTGTGCAATTCGCCGTCTATTTGACGGATCCGCAATTTGGCCAGAAAATCGTCTACAATG

GACGTGTGCGTGGGTCCAAAGGACGTTCAACCATTTCTGTGAAGGGTAACCCTAACAACGTTGTCGTGGGGAGCGTTGT

AGTAACCAAGACCGGTGCGGACCCGATGCAACAGACGGACTTGGAATCAATTGTGGGTATAATAGCACAGCGTGCTTGT

GATCAACTTGATACTATTGTTGTCGAGGATTGCCTGAATGGTGACGCCACTGACTCATGTCAATGTGATGAGGGATATG

GTGGAGCTAGATGTGAGACAAAGTGTATGAACGGTGATGTGGTGTCAACATCGCCATATAATGGACAAATTTGCGACTG

CGAACTAGGGTTTGGCGGATCTGATTGTAGTATGGAATCGGATTCATACCACTTCATGGCTAAAGCACAAACAGGGGCT

TTGAAATGCCAGGCTCTTTTTAATGAGCATACGGAATATTTATACGATGAACCAGGAGATAATCCATCTTCCATTAACC

CGTTCATGGATGAGAATTTACAAGATTATATTGGGGCGATTGAGATCTCTGGAAACATCCAATCGACAGTCCTGCAATT

CGGAGGCGATTACTCAGACTCCCACAACGCGTCACTAGAAAGGACAAACGACATGCTATTTGCTGCAAAGGTTGAAAAA

GAATATGATTTCGACTTCAAGCTGGAGATAGAAACGGTATATACAACAACAGTAACCTCAGAACAGCACACCCATCAAA

ATGGCACGCTCGGATATTGTGATTATCACTATCACATAGTCCAGGAGTACAGAGACGGCGTTAATCAAGCAGCAGCAGA

GATTTTTTTCGTCACCGGAGTGTCTGGCGTATCTGTGATGCCGGCTGCCGTAGTCAGAGACCCTGCTAGTGTTTCCAGT

GTAAATAAAAGGAATTCGTATTCAGCCAATGGTAACGATGTCGAAGAAATTATGATCGGGTTTACGTTGATTGGAACTG

TCGACTTGAAGTA

>HM9AYx1225F1_sec_allele_2

GAAATGTTGCTGAAAATATTCTGTCTTCCTTGAGCAATGACGACATTACTGCTATCCAGACCGGCTACGATTCTCCGTT

TGTATTTTCTGGAATATTATTCGCCGGATCGCAAATCGTCTATTTGGAAAACCTGGTGCAAAGGCTGAAATCTAACGTT

CCCTTATTATCTCCATCGGACTCTGGCGAATTCGAGGGATCAGAGTATGCTATCAGGACAGGATGGCCTTATATTCATG

TCGCTTACTTCAATAATTCGGACCGATCACGCGAGAGTGTTTCAGCTGGTATCAAAGCTACCCCAAGTGGCCTTACCTT

TAGCACACCAATCAACGCTACAGGAACCATTGATTCTTGCACCTACTCAGCTTATACCGTGCTGTCAGTGAAGGATGAG

GCAACAAGTTCAGTGGAATTTGTGCATTTCGCCGTCTATGTGACGGATGCGGACCCACAATTTGGCCGGAAAATCGTCT

ACAATGGACGTGTTCACGGGTCCAAAGGACGTTCAGCCATTTCCGTGAAGGGAAACCCCAACAACGTTGTCGTTGGGAG

CGTTGTATTAAGCAAGACCGGTGCGGACCCGATGCAACAGACGAATTTGGAAGCAATTGTGCGTATAATAGCACAGCGT

GCTTGTGATCAACTTGATACTATtGTTGTCGAGGAtTGCCTGAATGGTGAGGTCACTGACACATGTCAATGTGATGAGG

GATATGGTGGAGCTAGATGTGAGACAAAGTGTATGAACGGTGATGTGGTGTCAACATCGCCGTATAATGGACAAATTTG

CGACTGCGAGCTAGGATTTGGTGGATCTGATTGTAGTATGGAATCGGATTCATACCACTTCATGGCTAAAGCACAAACA

GGGGCTTTGAAATGTCAGGCTCTCTTTAATGACCATACGGGATATTTATACGATGAACCAGGAGCTAATCCATCTTCGA

TAACCCCGTTCATGGATCAGAATTTACAAAACTATATAGGGGCGATTGAGATCTCTGGAAACATCCAATCGACAGTCCT

GCAATTCGGAGGCGATTACTCAGACTCCCACAACGCGTCACTAGAAAGGACAAACGACATGCTATTTGCTGCAAAGGTT

GAAAAAGAATATGATTTCGACTTCAAGCTGGAGATAGAAACGGTATATACAACAACAGTAACCTCAGAACAGCACACCT

ATCAAAATGGCACACTCGGATATTGTGATTATCACTATCACATAGTCCAGGAGTACAGAGACGGCGTTAATCAAGCAGC

AGCAGAGGTTTTTTTCGTCACCGGAGTGTCTGGcGTATCTGTGATGCCGGCTGCTGTAGTCAGAGACCCTGCTAGTGTT

TCCAGTGTAAATAAAAGGAATTCGTATTCAGCCAATGGTAACGATGTCGAAGAAATTATGATCGGGTTTACGTTGATTG

GAACTGTCGACTTGAAGTA

>SC27f_sec_allele_1

GAAATGTTGCTGAAAATATTCTGTCTTCCTTGAGCAATGACGACATTACTGCTATCCAGACCGGCTACGATTCTCCGTT

GGTATTTTCTGGAATATTATTCGCCGGATCGCAAATCGTCTATTTGGAAAACCTGGTGCAAAGGCTGAAATCTAACGTT

CCCTTATTATCTCCATCGGACTCTGGCGAATTTGAGGGATCAGAGTATGCTGTCAGGACAGGATGGCCTTATATTCATA

TCGCTTACTTCAATAATTCGGACCGATCGCGCGAGAGTGTTTCAGCTGGTATCAAAACTACCCCAAGTGGCCTTACCTT

TAGCACACCAATCAACACTACAGGAACCATTGATTCTTGTACCTACTCAGCTTATACCGTGCTGTCACTGAAGGAAGAC

GAAACAAGTTCAGTGGAATTTGTGCAATTCGCCGTCTATTTGACGGATCCGCAATTTGGCCAGAAAATCGTCTACAATG

GACGTGTGCGTGGGTCCAAAGGACGTTCAACCATTTCTGTGAAGGGTAACCCTAACAACGTTGTCGTGGGGAGCGTTGT

AGTAACCAAGACCGGTGCGGACCCGATGCAACAGACGGACTTGGAATCAATTGTGGGTATAATAGCACAGCGTGCTTGT

GATCAACTTGATACTATTGTTGTCGAGGATTGCCTGAATGGTGACGCCACTGACTCATGTCAATGTGATGAGGGATATG

GTGGAGCTAGATGTGAGACAAAGTGTATGAACGGTGATGTGGTGTCAACATCGCCATATAATGGACAAATTTGCGACTG

CGAACTAGGGTTTGGCGGATCTGATTGTAGTATGGAATCGGATTCATACCACTTCATGGCTAAAGCACAAACAGGGGCT

TTGAAATGCCAGGCTCTTTTTAATGAGCATACGGAATATTTATACGATGAACCAGGAGATAATCCATCTTCCATTAACC

CGTTCATGGATGAGAATTTACAAGATTATATTGGGGCGATTGAGATCTCTGGAAACATCCAATCGACAGTCCTGCAATT

CGGAGGCGATTACTCAGACTCCCACAACGCGTCACTAGAAAGGACAAACGACATGCTATTTGCTGCAAAGGTTGAAAAA

GAATATGATTTCGACTTCAAGCTGGAGATAGAAACGGTATATACAACAACAGTAACCTCAGAACAGCACACCCATCAAA

ATGGCACGCTCGGATATTGTGATTATCACTATCACATAGTCCAGGAGTACAGAGACGGCGTTAATCAAGCAGCAGCAGA

GATTTTTTTCGTCACCGGAGTGTCTGGCGTATCTGTGATGCCGGCTGCCGTAGTCAGAGACCCTGCTAGTGTTTCCAGT

GTAAATAAAAGGAATTCGTATTCAGCCAATGGTAACGATGTCGAAGAAATTATGATCGGGTTTACGTTGATTGGAACTG

TCGACTTGAAGTA

>SC27f_sec_allele_2

GAAATGTTGCTGAAAATATTCTGTCTTCCTTGAGCAATGCCGACATCAATGCTATCCACAGCGGCTACGATTCTCCATT

TGTATTTCCTGGAATAATATTCGCCGGATCGCAAATCGTCTATTTGGAAAACCTGGTGCAAAGGCTGAAATCTAATGCT

CCCTTATCATCTCCATTGGAGTCTGGCGAATTCGAGGGATCAGAGTATGCTGTCAGGACAGGATGGCCTTATATTCATA

TCGCTTACTTCAATAATTCGGACCGATCACGCGAGAGTGTTTCAGCTGCTATCAAAGCTACCCCAAGTGGCCTTACCTT

TAGCACACCAATCAACGCTACAGGAACCATTGATTCTTGCACCTACTCAGCTTATACCTTGCTGTCAGTGAAGGAAGAG

GCAACAAGTTCAGTAGAATTTGTGCATTTCGCCGTCTATGTGACGGATGCGGATCCACAATTTGGCCGGAAAATCGTCT

ACAATGGCCGTGTTCACGGGTCCAAAGGACGTTCAACCATTTCCGTGAAGGGAAACCCCAACAACGTCGTCGTTGGGAG

CGTTGTATTAAGCAAGACCGGTGCGGACCCGATGCAACAGACGGATTTGGAAGCAATTGTGCGTATAATAGCACAGCGT

GCTTGTGAaCAACTTGATAaTATtGTTGTCGAGGAtTGCCTGAATGGTGAGGTCACTGACACATGTCAATGTGATGAtG

GATATGGTGGAGCTAGATGTGAGACAAAATGTATGAACGGTGATGTGGTGTCAACATCGCCGTATAATGGACAAATTTG

CGACTGCGgGCTAGGATTTGGCGGATCTGATTGTAGTATGGAATCGGATTCATACCACTTCATGGCTAAAGCACGAACA

GGGGCTTTGAAATGTCGGGCTCTCTTTAATGACCATACGGAATATTTATACGATGAACCAGGAGCTAATCCATCTTCGA

TAACCCCGTTCATGGATCAGAATTTACAAAATTATATAGGGGCGATTGAGATCTCTGGAAACATCCAATCAACAGTCTT

GCAATTCGGAGGCGATTACTCAGACTCCCACAACGCGTCACTAGAAAGGACAAACGACATGCTATTTGCTGCAaaGGTT

GAAAAAGAATATGATTTCGACTTCAGGCTGGAGATAGAAACGGTATACGCAACAACGGTAACCTCAGAACAGCACACCT

ATCAAAATGGCACACTCGGATATTGTGATTATCACTATCACATAGTCCAGGAGTACAGAGACGGCGTGAATCAAGCTGC

AGCAGAGATTTTTTTCGTCACCGGAGTGTCTGGCGTATCTGTGATGCCGGCTGCTGTAGTCAGAGACCCTGCTAGTGTT

TCCAGTGTAAATAAAAGGAATTCATATTCAGCCAATGGTAACGATGTCGAAGAAATTATGATCGGGTTTACGTTGATTG

GAACTGTCGACTTGAAGTA

>SC32e_sec_allele_1

GAAATGTTGCTGAAAATATTCTGTCTTCCTTGAGCAATGACGACATTACTGCTATCCAGACCGGCTACGATTCTCCGTT

TGTATTTTCTGGAATATTATTCGCCGGATCGCAAATCGTCTATTTGGAAAACCTGGTGCAAAGGCTGAAATCTAACGTT

CCCTTATTATCTCCATCGGACTCTGGCGAATTCGAGGGATCAGAGTATGCTATCAGGACAGGATGGCCTTATATTCATG

TCGCTTACTTCAATAATTCGGACCGATCACGCGAGAGTGTTTCAGCTGGTATCAAAGCTACCCCAAGTGGCCTTACCTT

TAGCACACCAATCAACGCTACAGGAACCATTGATTCTTGCACCTACTCAGCTTATACCGTGCTGTCAGTGAAGGATGAG

GCAACAAGTTCAGTGGAATTTGTGCATTTCGCCGTCTATGTGACGGATGCGGACCCACAATTTGGCCGGAAAATCGTCT

ACAATGGACGTGTTCACGGGTCCAAAGGACGTTCAGCCATTTCCGTGAAGGGAAACCCCAACAACGTTGTCGTTGGGAG

CGTTGTATTAAGCAAGACCGGTGCGGACCCGATGCAACAGACGAATTTGGAAGCAATTGTGCGTATAATAGCACAGCGT

GCTTGTGATCAACTTGATACTATTGTTGTCGAGGATTGCCTGAATGGTGAGGTCACTGACACATGTCAATGTGATGAGG

GATATGGTGGAGCTAGATGTGAGACAAAGTGTATGAACGGTGATGTGGTGTCAACATCGCCGTATAATGGACAAATTTG

CGACTGCGAGCTAGGATTTGGTGGATCTGATTGTAGTATGGAATCGGATTCATACCACTTCATGGCTAAAGCACAAACA

GGGGCTTTGAAATGTCAGGCTCTCTTTAATGACCATACGGGATATTTATACGATGAACCAGGAGCTAATCCATCTTCGA

TAACCCCGTTCATGGATCAGAATTTACAAAACTATATAGGGGCGATTGAGATCTCTGGAAACATCCAATCGACAGTCCT

GCAATTCGGAGGCGATTACTCAGACTCCCACAACGCGTCACTAGAAAGGACAAACGACATGCTATTTGCTGCAAAGGTT

GAAAAAGAATATGATTTCGACTTCAAGCTGGAGATAGAAACGGTATATACAACAACAGTAACCTCAGAACAGCACACCT

ATCAAAATGGCACACTCGGATATTGTGATTATCACTATCACATAGTCCAGGAGTACAGAGACGGCGTTAATCAAGCAGC

AGCAGAGGTTTTTTtCGTCACCGGAGTGTCTGGCGTATCTGTGATGCCGGCTGCTGTAGTCAGAGACCCTGCTAGTGTT

TCCAGTGTAAATAAAAGGAATTCGTATTCAGCCAATGGTAACGATGTCGAAGAAATTATGATCGGGTTTACGTTGATTG

GAACTGTCGACTTGAAGTA

>SC32e_sec_allele_2

GAAATGTTGCTGAAAATATTCTGTCTTCCTTGAGCAATGCCGACATTACTGCTATCAACTACGATTCTCCATTTGTATT

TCCTGGACTAATATTCGCCGGATCGCAAATCGTCTATTTGGAAAACGTGGTGCAAAGGCTGAAATCTAATGTTCCCTTA

TCATCTCCATTGGACTCTGGCGAATTTGAGGGATCAGAGTATGCTGTCAGGACAGGATGGCCTTATATTCATATCGCTT

ACTTCAATAACTCGGACCGATCACGCGACAGTGTTTCAGCTGGTATCAAAACTACCCCAAGTGGCCTTACCTTTAGCAC

ACCAATCAACACTACAGGAACCATTGATTCTTGTACCTACTCAGCTTATACCGTGCTGTCAGCGAAGGAAGAGGAAACA

AGTTCAGTGGAATTTGTGCAATTCGCCGTCTATTTGACGGATGCGGATCCGCAATTTGGCCAGAAAATCGTCTACAATG

CACGTGTGCGTGGGTCCAAAGGACGTTCAACCATTTCTGTGAAGGGTAACCCTAACAACGTTGTCGTGGGGAGCGTTGT

AGTAACCAAGACCGGTGTGGACCCAATGCAACAGACGGACTTGGAAGCAATTGTGGGTATAATAGCACAGCGTGCTTGT

GATCAACTTGATACTATTGTTGTCGAGGATTGCCTGAATGGTGAGGCCACTGACTCATGTCAATGTGATGAGGGATATG

GTGGAGCTAGATGTGAGACAAAGTGTATGAACGGTGATGTGGTGTCAACATCGCCATATAATGGACAAATTTGCGACTG

CGAACTAGGATTTGGTGGATCTGATTGTAGTATGGAATCGGATTCATACCACTTCATGGCTGAAGCACAAACAGGGGCT

TTGAGATGTCAGGCTCTCTTTAATGACCATACGGGATATTTATACGATGAACCAGGGGCTAATCCATCTTCGATAACCC

CGTTCATGGATCGGAATTTACAAAATTATATAGGGGCGATTGAGATCTCTGGAAACATCCAATCAACAGTCCTGCAATT

CGGAGGCGATTACTCAGACTCCCACAACGCGTCACTAGAAAGGACAAACGACATGCTGTTTGCTGCAAAGGTTGAAAAA

GAATATGATTTCGACTTTAAGCTGGAGATAGAAACGGTATATGCAACAACAGTAACCTCAGAACAGCACACCTATCAAA

ATGGCACGCTCGGATATTGTGATTATCACTATCACATAGTCCAGGAGTACAGAGACGGCGTTAATCAAGCAGCAGCAGA

GATTTTTTTCGTCACCGGAGTGTCTGGCGTATCTGTGATGCCGGCTGCTGTAGTCAGAGACCCTGCTAGTGTTTCCAGT

GTAAATAAAAGGAATTCGTATTCAGCCAATGGTAACGATGTCGAAGAAATTATGATCGGGTTTACGTTGATTGGAACTG

TCGACTTGAAGTA

>SB1_SEC_allele_1

GAAATCTAACGTTCCCTTATTATCTCCATCGGACTCTGGCGAATTTGAGGGATCAGAGTATGCTGTCAGGACAGGATGG

CCTTATATTCATATCGCTTACTTCAATAATTCGGACCGATCGCGCGAGAGTGTTTCAGCTGGTATCAAAGCTTCCTCAA

GTGGCCTTACCTTTAGCACACCAATAAACACTACAGGAACCGTTGATTCTTGTACCTACTCAGCTCATACCCTGCTGTC

AGTGAAGGATGAGGCAACAAGTTCAGTGGAATTTGTGCAATTCACCGTCTATTTGACGGAGGCGGATCCACAAGTAACA

AAAATCATCTACAATAGCCGTGTGCATGGGTCCATAGGACGTTCAACCATTTCCGTGAAGGGAAACCCCAACAACGTTG

TCGTTGGGAGCGTTGTATTAAGCAAGACCGGTGCGGACCCGATGCAACAGACGGATTTGGAAGCAATTGTGCGTATAAT

AGCACAGCGTGCTTGTGATCGACTTGATACTATTGTTGTCGAGGATTGCCTGAATGGTGAGGTCACTGACTCATGTCAA

TGTGATGAGGGATATGGTGGAGCTAGATGTGAGACAAAGTGTATGAACGGTGATGTGGTGTCAACATCGCCATATAATG

GACAAATTTGCGACTGCGAACTAGGATTTGGTGGATCTGATTGTAGTAAGGAATCGGATTCATACCACTTTATGGCTAA

AGCACAAACAGGAGCTTTGAAATGTCAGGCTCTCTTTAATGACCATACGGGATATTTATACGATGAACCAGGAGCTA

>SB1_SEC_allele_2

GAAATCTAACGTTCCCTTATTATCTCCATTGGACTCTGGCGAATTTGAGGGATCAGAGTATGCTGTCAGGACAGGATGG

CCTTATATTCATATCGCTTACTTCAATAACTCGGACCGATCACGCGACAGTGTTTCAGCTGGTATCAAAACTACCCCAA

GTGGCCTTACCTTTAGCACACCAATCAACACTACAGGAACCATTGATTCTTGTACCTACTCAGCTTATACCGTGCTGTC

AGCGAAGGAAGAGGAAACAAGTTCAGTGGAATTTGTGCAATTCGCCGTCTATTTGACGGATGCGGATCCGCAATTTGGC

CAGAAAATCGTCTACAATGCACGTGTGCGTGGGTCCAAAGGACGTTCAACCATTTCTGTGAAGGGTAACCCTAACAACG

TTGTCGTGGGGAGCGTTGTAGTAACCAAGACCGGTGTGGACCCAATGCAACAGACGGACTTGGAAGCAATTGTGGGTAT

AATAGCACAGCGTGCTTGTGATCAACTTGATACTATTGTTGTCGAGGATTGCCTGAATGGTGAGGCCACTGACTCATGT

CAATGTGATGAGGGATATGGTGGAGCTAGATGTGAGACAAAGTGTATGAACGGTGATGTGGTGTCAACATCGCCATATA

ATGGACAAATTTGCGACTGCGAACTAGGATTTGGTGGATCTGATTGTAGTATGGAATCGGATTCATACCACTTCATGGC

TGAAGCACAAACAGGGGCTTTGAGATGTCAGGCTCTCTTTAATGACCATACGGGATATTTATACGATGAACCAGGAGCT

A

>SB2_SEC_allele_1

GAAATCTAACGTTCCCTTATTATCTCCATCGGACTCTGGCGAATTTGAGGGATCAGAGTATGCTGTCAGGACAGGATGGC

CTTATATTCATATCGCTTACTTCAATAATTCGGACCGATCGCGCGAGAGTGTTTCAGCTGGTATCAAAACTACCCCAAGT

GGCCTTACCTTTAGCACACCAATCAACACTACAGGAACCGTTGATTCTTGTACCTACTCAGCTCATACCCTGCTGTCAGT

GAAGGATGAGGCAACAAGTTCAGTGGAATTTGTGCAATTCACCGTCTATTTGACGGAGGCGGATCCACAAAAAATCATCT

ACAATAGCCGTGTGCATGGGTCCATAGGACGTTCAACCATTTCCGTGAAGGGAAACCCCAACAACGTTGTCGTTGGGAGC

GTTGTATTAAGCAAGACCGGTGCGGACCCGATGCAACAGACGGATTTGGAAGCAATTGTGCGTATAATAGCACAGCGTGC

TTGTGATCGACTTGATACTATTGTTGTCGAGGATTGCCTGAATGGTGAGGTCACTGACACATGTCAATGTGATGATGGAT

ATGGTGGAGCTAGATGTGAGACAAAGTGTATGAACGGTGATGTGGTGTCAACATCGCCATATAATGGACAAATTTGCGAC

TGCGAGCTAGGATTTGGTGGATCTGATTGTAGTAAGGAATCGGATTCATACCACTTTATGGCTAAAGCACAAACAGGAGC

TTTGAAATGTCAGGCTCTCTTTAATGACCATACGGGATATTTATACGATGAACCAGGAGCTA

>SB2_SEC_allele_2

GAAATCTAACGTTCCCTTATTATCTCCATCGGACTCTGGCGAATTTGAGGGATCAGAGTATGCTGTCAGGACAGGATGG

CCTTATATTCATATCGCTTACTTCAATAATTCGGACCGATCGCGCGAGAGTGTTTCAGCTGGTATCAAAACTACCCCAA

GTGGCCTTACCTTTAGCACACCAATCAACACTACAGGAACCATTGATTCTTGTACCTACTCAGCTTATACCGTGCTGTC

AGCGAAGGAAGAGGAAACAAGTTCAGTGGAATTTGTGCAATTCGCCGTCTATTTGACGGATGCGGATCCGCAATTTGGC

CAGAAAATCGTCTACAATGCACGTGTGCGTGGGTCCAAAGGACGTTCAACCATTTCTGTGAAGGGTAACCCTAACAACG

TTGTCGTGGGGAGCGTTGTAGTAACCAAGACCGGTGTGGACCCAATGCAACAGACGGACTTGGAAGCAATTGTGGGTAT

AATAGCACAGCGTGCTTGTGATCAACTTGATACTATTGTTGTCGAGGATTGCCTGAATGGTGAGGCCACTGACTCATGT

CAATGTGATGAGGGATATGGTGGAGCTAGATGTGAGACAAAGTGTATGAACGGTGATGTGGTGTCAACATCGCCATATA

ATGGACAAATTTGCGACTGCGAACTAGGATTTGGTGGATCTGATTGTAGTATGGAATCGGATTCATACCACTTCATGGC

TGAAGCACAAACAGGGGCTTTGAGATGTCAGGCTCTCTTTAATGACCATACGGGATATTTATACGATGAACCAGGAGCT

A

>SB3_SEC_allele_1

GAAATCTAACGTTCCCTTATTATCTCCATCGGACTCTGGCGAATTTGAGGGATCAGAGTATGCTGTCAGGACAGGATGGC

CTTATATTCATATCGCTTACTTCAATAATTCGGACCGATCGCGCGAGAGTGTTTCAGCTGGTATCAAAACTACCCCAAGT

GGCCTTACCTTTAGCACACCAATCAACACTACAGGAACCGTTGATTCTTGTACCTACTCAGCTCATACCCTGCTGTCAGT

GAAGGATGAGGCAACAAGTTCAGTGGAATTTGTGCAATTCACCGTCTATTTGACGGAGGCGGATCCACAAAAAATCATCT

ACAATAGCCGTGTGCATGGGTCCATAGGACGTTCAACCATTTCCGTGAAGGGAAACCCCAACAACGTTGTCGTTGGGAGC

GTTGTATTAAGCAAGACCGGTGCGGACCCGATGCAACAGACGGATTTGGAAGCAATTGTGCGTATAATAGCACAGCGTGC

TTGTGATCGACTTGATACTATTGTTGTCGAGGATTGCCTGAATGGTGAGGTCACTGACACATGTCAATGTGATGATGGAT

ATGGTGGAGCTAGATGTGAGACAAAGTGTATGAACGGTGATGTGGTGTCAACATCGCCATATAATGGACAAATTTGCGAC

TGCGAGCTAGGATTTGGTGGATCTGATTGTAGTAAGGAATCGGATTCATACCACTTTATGGCTAAAGCACAAACAGGAGC

TTTGAAATGTCAGGCTCTCTTTAATGACCATACGGGATATTTATACGATGAACCAGGAGCTA

>SB3_SEC_allele_2

GAAATCTAACGTTCCCTTATTATCTCCATTGGACTCTGGCGAATTTGAGGGATCAGAGTATGCTGTCAGGACAGGATGGC

CTTATATTCATATCGCTCACTTCAATAATTCGGACCGATCGCGCGAGAGTGTTTCAGCTGGTATCAAAGCTACCCCAAGT

GGCCTTACCTTTAGCACACCAATCAACACTACAGGAACCATTGATTCTTGTACCTACTCAGCTTATACCGTGCTGTCAGT

GAAGGAAGAGGCAACAAGTTCAGTGGAATTTGTGCAATTCGCCGTCTTTTTGACGGATGCGGATCCACAATTTGGCCGGA

AAATCGTCTACAATGGCCGTGTTCACGGGTCCAAAGGACGTTCAACCATTTCCGTGAAGGGAAACCCCAACAACGTCGTC

GTTGGGAGCGTTGTATTAACCAAGACCGGTGCGGACCCGATGCAACAGACGGATTTGGAAGCAATTGTGCGTATAATAGC

ACAGCGTGCCTGTGATCAACTTGATACTATTGTTGTCGAGGATTGCCTGAATGGTGAGCTCACAGACACATGTCAATGTG

ATGAGGGATATGGTGGAGCTAGATGTGAGACAAAGTGTATGAACGGTGATGTGGTGTCAACATCGCCATATAATGGACAA

ATTTGCAACTGCGAACTAGGATTTGGTGGATCTGATTGTAGTATGGAATCGGATTCATACCACTTCATGGCTAGAGCACA

AACAGGGGCTTTGAAATGTCAGGCTCTCTTTAATGACCATACGGGATATTTATACGATGAACCAGGAGCTA

>SB4_SEC_allele_1

GAAATCTAACGTTCCCTTATTATCTCCATCGGACTCTGGCGAATTTGAGGGATCAGAGTATGCTGTCAGGACAGGATGG

CCTTATATTCATATCGCTTACTTCAATAATTCGGACCGATCGCGCGAGAGTGTTTCAGCTGGTATCAAAACTACCCCAA

GTGGCCTTACCTTTAGCACACCAATCAACACTACAGGAACCATTGATTCTTGTACCTACTCAGCTTATACCGTGCTGTC

AGCGAAGGAAGAGGAAACAAGTTCAGTGGAATTTGTGCAATTCGCCGTCTATTTGACGGATGCGGATCCGCAATTTGGC

CAGAAAATCGTCTACAATGCACGTGTGCGTGGGTCCAAAGGACGTTCAACCATTTCTGTGAAGGGTAACCCTAACAACG

TTGTCGTGGGGAGCGTTGTAGTAACCAAGACCGGTGTGGACCCAATGCAACAGACGGACTTGGAAGCAATTGTGGGTAT

AATAGCACAGCGTGCTTGTGATCAACTTGATACTATTGTTGTCGAGGATTGCCTGAATGGTGAGGCCACTGACTCATGT

CAATGTGATGAGGGATATGGTGGAGCTAGATGTGAGACAAAGTGTATGAACGGTGATGTGGTGTCAACATCGCCATATA

ATGGACAAATTTGCGACTGCGAACTAGGATTTGGTGGATCTGATTGTAGTATGGAATCGGATTCATACCACTTCATGGC

TGAAGCACAAACAGGGGCTTTGAGATGTCAGGCTCTCTTTAATGACCATACGGGATATTTATACGATGAACCAGGAGCT

A

>SB4_SEC_allele_2

GAAATCTAACGTTCCCTTATTATCTCCATTGGACTCTGGCGAATTTGAGGGATCAGAGTATGCTGTCAGGACAGGATGGC

CTTATATTCATATCGCTCACTTCAATAATTCGGACCGATCGCGCGAGAGTGTTTCAGCTGGTATCAAAGCTACCCCAAGT

GGCCTTACCTTTAGCACACCAATCAACACTACAGGAACCATTGATTCTTGTACCTACTCAGCTTATACCGTGCTGTCAGT

GAAGGAAGAGGCAACAAGTTCAGTGGAATTTGTGCAATTCGCCGTCTTTTTGACGGATGCGGATCCACAATTTGGCCGGA

AAATCGTCTACAATGGCCGTGTTCACGGGTCCAAAGGACGTTCAACCATTTCCGTGAAGGGAAACCCCAACAACGTCGTC

GTTGGGAGCGTTGTATTAACCAAGACCGGTGCGGACCCGATGCAACAGACGGATTTGGAAGCAATTGTGCGTATAATAGC

ACAGCGTGCCTGTGATCAACTTGATACTATTGTTGTCGAGGATTGCCTGAATGGTGAGCTCACAGACACATGTCAATGTG

ATGAGGGATATGGTGGAGCTAGATGTGAGACAAAGTGTATGAACGGTGATGTGGTGTCAACATCGCCATATAATGGACAA

ATTTGCAACTGCGAACTAGGATTTGGTGGATCTGATTGTAGTATGGAATCGGATTCATACCACTTCATGGCTAGAGCACA

AACAGGGGCTTTGAAATGTCAGGCTCTCTTTAATGACCATACGGGATATTTATACGATGAACCAGGAGCTA

>SB6_SEC_allele_1

GAAATCTAACGTTCCCTTATTATCTCCATCGGACTCTGGCGAATTTGAGGGATCAGAGTATGCTGTCAGGACAGGATGG

CCTTATATTCATATCGCTTACTTCAATAATTCGGACCGATCGCGCGAGAGTGTTTCAGCTGGTATCAAAGCTTCCTCAA

GTGGCCTTACCTTTAGCACACCAATAAACACTACAGGAACCGTTGATTCTTGTACCTACTCAGCTCATACCCTGCTGTC

AGTGAAGGATGAGGCAACAAGTTCAGTGGAATTTGTGCAATTCACCGTCTATTTGACGGAGGCGGATCCACAAGTAACA

AAAATCATCTACAATAGCCGTGTGCATGGGTCCATAGGACGTTCAACCATTTCCGTGAAGGGAAACCCCAACAACGTTG

TCGTTGGGAGCGTTGTATTAAGCAAGACCGGTGCGGACCCGATGCAACAGACGGATTTGGAAGCAATTGTGCGTATAAT

AGCACAGCGTGCTTGTGATCGACTTGATACTATTGTTGTCGAGGATTGCCTGAATGGTGAGGTCACTGACTCATGTCAA

TGTGATGAGGGATATGGTGGAGCTAGATGTGAGACAAAGTGTATGAACGGTGATGTGGTGTCAACATCGCCATATAATG

GACAAATTTGCGACTGCGAACTAGGATTTGGTGGATCTGATTGTAGTAAGGAATCGGATTCATACCACTTTATGGCTAA

AGCACAAACAGGAGCTTTGAAATGTCAGGCTCTCTTTAATGACCATACGGGATATTTATACGATGAACCAGGAGCTA

>SB6_SEC_allele_2

GAAATCTAACGTTCCCTTATTATCTCCATCGGACTCTGGCGAATTTGAGGGATCAGAGTATGCTGTCAGGACAGGATGG

CCTTATATTCATATCGCTTACTTCAATAATTCGGACCGATCGCGCGAGAGTGTTTCAGCTGGTATCAAAACTACCCCAA

GTGGCCTTACCTTTAGCACACCAATCAACACTACAGGAACCATTGATTCTTGTACCTACTCAGCTTATACCGTGCTGTC

ACTGAAGGAAGACGAAACAAGTTCAGTGGAATTTGTGCAATTCGCCGTCTATTTGACGGATCCGCAATTTGGCCAGAAA

ATCGTCTACAATGGACGTGTGCGTGGGTCCAAAGGACGTTCAACCATTTCTGTGAAGGGTAACCCTAACAACGTTGTCG

TGGGGAGCGTTGTAGTAACCAAGACCGGTGCGGACCCGATGCAACAGACGGACTTGGAATCAATTGTGGGTATAATAGC

ACAGCGTGCTTGTGATCAACTTGATACTATTGTTGTCGAGGATTGCCTGAATGGTGACGCCACTGACTCATGTCAATGT

GATGAGGGATATGGTGGAGCTAGATGTGAGACAAAGTGTATGAACGGTGATGTGGTGTCAACATCGCCATATAATGGAC

AAATTTGCGACTGCGAACTAGGGTTTGGCGGATCTGATTGTAGTATGGAATCGGATTCATACCACTTCATGGCTAAAGC

ACAAACAGGGGCTTTGAAATGCCAGGCTCTCTTTAATGACCATACGGGATATTTATACGATGAACCAGGAGCTA

**fuhc secreted protein sequences**

>Hm9aY x 1225F1 allele 1

NVAENILSSLSNDDITAIQTGYDSPLVFSGILFAGSQIVYLENLVQRLKSNVPLLSPSDSGEFEGSEYAVRTGWPYIHIA

YFNNSDRSRESVSAGIKTTPSGLTFSTPINTTGTIDSCTYSAYTVLSLKEDETSSVEFVQFAVYLTDPQFGQKIVYNGRV

RGSKGRSTISVKGNPNNVVVGSVVVTKTGADPMQQTDLESIVGIIAQRACDQLDTIVVEDCLNGDATDSCQCDEGYGGAR

CETKCMNGDVVSTSPYNGQICDCELGFGGSDCSMESDSYHFMAKAQTGALKCQALFNEHTEYLYDEPGDNPSSINPFMDE

NLQDYIGAIEISGNIQSTVLQFGGDYSDSHNASLERTNDMLFAAKVEKEYDFDFKLEIETVYTTTVTSEQHTHQNGTLGY

CDYHYHIVQEYRDGVNQAAAEIFFVTGVSGVSVMPAAVVRDPASVSSVNKRNSYSANGNDVEEIMIGFTLIGTVDLK

> Hm9aY x 1225F1 allele 2

NVAENILSSLSNDDITAIQTGYDSPFVFSGILFAGSQIVYLENLVQRLKSNVPLLSPSDSGEFEGSEYAIRTGWPYIHVA

YFNNSDRSRESVSAGIKATPSGLTFSTPINATGTIDSCTYSAYTVLSVKDEATSSVEFVHFAVYVTDADPQFGRKIVYNG

RVHGSKGRSAISVKGNPNNVVVGSVVLSKTGADPMQQTNLEAIVRIIAQRACDQLDTIVVEDCLNGEVTDTCQCDEGYGG

ARCETKCMNGDVVSTSPYNGQICDCELGFGGSDCSMESDSYHFMAKAQTGALKCQALFNDHTGYLYDEPGANPSSITPFM

DQNLQNYIGAIEISGNIQSTVLQFGGDYSDSHNASLERTNDMLFAAKVEKEYDFDFKLEIETVYTTTVTSEQHTYQNGTL

GYCDYHYHIVQEYRDGVNQAAAEVFFVTGVSGVSVMPAAVVRDPASVSSVNKRNSYSANGNDVEEIMIGFTLIGTVDLK

>SC32e allele 1

NVAENILSSLSNDDITAIQTGYDSPFVFSGILFAGSQIVYLENLVQRLKSNVPLLSPSDSGEFEGSEYAIRTGWPYIHVA

YFNNSDRSRESVSAGIKATPSGLTFSTPINATGTIDSCTYSAYTVLSVKDEATSSVEFVHFAVYVTDADPQFGRKIVYNG

RVHGSKGRSAISVKGNPNNVVVGSVVLSKTGADPMQQTNLEAIVRIIAQRACDQLDTIVVEDCLNGEVTDTCQCDEGYGG

ARCETKCMNGDVVSTSPYNGQICDCELGFGGSDCSMESDSYHFMAKAQTGALKCQALFNDHTGYLYDEPGANPSSITPFM

DQNLQNYIGAIEISGNIQSTVLQFGGDYSDSHNASLERTNDMLFAAKVEKEYDFDFKLEIETVYTTTVTSEQHTYQNGTL

GYCDYHYHIVQEYRDGVNQAAAEVFFVTGVSGVSVMPAAVVRDPASVSSVNKRNSYSANGNDVEEIMIGFTLIGTVDLK

>SC32e allele 2

NVAENILSSLSNADITAINYDSPFVFPGLIFAGSQIVYLENVVQRLKSNVPLSSPLDSGEFEGSEYAVRTGWPYIHIAYF

NNSDRSRDSVSAGIKTTPSGLTFSTPINTTGTIDSCTYSAYTVLSAKEEETSSVEFVQFAVYLTDADPQFGQKIVYNARV

RGSKGRSTISVKGNPNNVVVGSVVVTKTGVDPMQQTDLEAIVGIIAQRACDQLDTIVVEDCLNGEATDSCQCDEGYGGAR

CETKCMNGDVVSTSPYNGQICDCELGFGGSDCSMESDSYHFMAEAQTGALRCQALFNDHTGYLYDEPGANPSSITPFMDR

NLQNYIGAIEISGNIQSTVLQFGGDYSDSHNASLERTNDMLFAAKVEKEYDFDFKLEIETVYATTVTSEQHTYQNGTLGY

CDYHYHIVQEYRDGVNQAAAEIFFVTGVSGVSVMPAAVVRDPASVSSVNKRNSYSANGNDVEEIMIGFTLIGTVDLK

>SC27f allele 1

NVAENILSSLSNDDITAIQTGYDSPLVFSGILFAGSQIVYLENLVQRLKSNVPLLSPSDSGEFEGSEYAVRTGWPYIHIA

YFNNSDRSRESVSAGIKTTPSGLTFSTPINTTGTIDSCTYSAYTVLSLKEDETSSVEFVQFAVYLTDPQFGQKIVYNGRV

RGSKGRSTISVKGNPNNVVVGSVVVTKTGADPMQQTDLESIVGIIAQRACDQLDTIVVEDCLNGDATDSCQCDEGYGGAR

CETKCMNGDVVSTSPYNGQICDCELGFGGSDCSMESDSYHFMAKAQTGALKCQALFNEHTEYLYDEPGDNPSSINPFMDE

NLQDYIGAIEISGNIQSTVLQFGGDYSDSHNASLERTNDMLFAAKVEKEYDFDFKLEIETVYTTTVTSEQHTHQNGTLGY

CDYHYHIVQEYRDGVNQAAAEIFFVTGVSGVSVMPAAVVRDPASVSSVNKRNSYSANGNDVEEIMIGFTLIGTVDLK

>SC27f allele 2

NVAENILSSLSNADINAIHSGYDSPFVFPGIIFAGSQIVYLENLVQRLKSNAPLSSPLESGEFEGSEYAVRTGWPYIHIA

YFNNSDRSRESVSAAIKATPSGLTFSTPINATGTIDSCTYSAYTLLSVKEEATSSVEFVHFAVYVTDADPQFGRKIVYNG

RVHGSKGRSTISVKGNPNNVVVGSVVLSKTGADPMQQTDLEAIVRIIAQRACEQLDNIVVEDCLNGEVTDTCQCDDGYGG

ARCETKCMNGDVVSTSPYNGQICDCGLGFGGSDCSMESDSYHFMAKARTGALKCRALFNDHTEYLYDEPGANPSSITPFM

DQNLQNYIGAIEISGNIQSTVLQFGGDYSDSHNASLERTNDMLFAAKVEKEYDFDFRLEIETVYATTVTSEQHTYQNGTL

GYCDYHYHIVQEYRDGVNQAAAEIFFVTGVSGVSVMPAAVVRDPASVSSVNKRNSYSANGNDVEEIMIGFTLIGTVDLK

>SB1_SEC_allele_1

KSNVPLLSPSDSGEFEGSEYAVRTGWPYIHIAYFNNSDRSRESVSAGIKASSSGLTFSTPINTTGTVDSCTYSAHTLLSV

KDEATSSVEFVQFTVYLTEADPQVTKIIYNSRVHGSIGRSTISVKGNPNNVVVGSVVLSKTGADPMQQTDLEAIVRIIAQ

RACDRLDTIVVEDCLNGEVTDSCQCDEGYGGARCETKCMNGDVVSTSPYNGQICDCELGFGGSDCSKESDSYHFMAKAQT

GALKCQALFNDHTGYLYDEPGA

>SB1_SEC_allele_2

KSNVPLLSPLDSGEFEGSEYAVRTGWPYIHIAYFNNSDRSRDSVSAGIKTTPSGLTFSTPINTTGTIDSCTYSAYTVLSA

KEEETSSVEFVQFAVYLTDADPQFGQKIVYNARVRGSKGRSTISVKGNPNNVVVGSVVVTKTGVDPMQQTDLEAIVGIIA

QRACDQLDTIVVEDCLNGEATDSCQCDEGYGGARCETKCMNGDVVSTSPYNGQICDCELGFGGSDCSMESDSYHFMAEAQ

TGALRCQALFNDHTGYLYDEPGA

>SB2_SEC_allele_1

KSNVPLLSPSDSGEFEGSEYAVRTGWPYIHIAYFNNSDRSRESVSAGIKTTPSGLTFSTPINTTGTVDSCTYSAHTLLSV

KDEATSSVEFVQFTVYLTEADPQKIIYNSRVHGSIGRSTISVKGNPNNVVVGSVVLSKTGADPMQQTDLEAIVRIIAQRA

CDRLDTIVVEDCLNGEVTDTCQCDDGYGGARCETKCMNGDVVSTSPYNGQICDCELGFGGSDCSKESDSYHFMAKAQTGA

LKCQALFNDHTGYLYDEPGA

>SB2_SEC_allele_2

KSNVPLLSPSDSGEFEGSEYAVRTGWPYIHIAYFNNSDRSRESVSAGIKTTPSGLTFSTPINTTGTIDSCTYSAYTVLSA

KEEETSSVEFVQFAVYLTDADPQFGQKIVYNARVRGSKGRSTISVKGNPNNVVVGSVVVTKTGVDPMQQTDLEAIVGIIA

QRACDQLDTIVVEDCLNGEATDSCQCDEGYGGARCETKCMNGDVVSTSPYNGQICDCELGFGGSDCSMESDSYHFMAEAQ

TGALRCQALFNDHTGYLYDEPGA

>SB3_SEC_allele_1

KSNVPLLSPSDSGEFEGSEYAVRTGWPYIHIAYFNNSDRSRESVSAGIKTTPSGLTFSTPINTTGTVDSCTYSAHTLLSV

KDEATSSVEFVQFTVYLTEADPQKIIYNSRVHGSIGRSTISVKGNPNNVVVGSVVLSKTGADPMQQTDLEAIVRIIAQRA

CDRLDTIVVEDCLNGEVTDTCQCDDGYGGARCETKCMNGDVVSTSPYNGQICDCELGFGGSDCSKESDSYHFMAKAQTGA

LKCQALFNDHTGYLYDEPGA

>SB3_SEC_allele_2

KSNVPLLSPLDSGEFEGSEYAVRTGWPYIHIAHFNNSDRSRESVSAGIKATPSGLTFSTPINTTGTIDSCTYSAYTVLSV

KEEATSSVEFVQFAVFLTDADPQFGRKIVYNGRVHGSKGRSTISVKGNPNNVVVGSVVLTKTGADPMQQTDLEAIVRIIA

QRACDQLDTIVVEDCLNGELTDTCQCDEGYGGARCETKCMNGDVVSTSPYNGQICNCELGFGGSDCSMESDSYHFMARAQ

TGALKCQALFNDHTGYLYDEPGA

>SB4_SEC_allele_1

KSNVPLLSPSDSGEFEGSEYAVRTGWPYIHIAYFNNSDRSRESVSAGIKTTPSGLTFSTPINTTGTIDSCTYSAYTVLSA

KEEETSSVEFVQFAVYLTDADPQFGQKIVYNARVRGSKGRSTISVKGNPNNVVVGSVVVTKTGVDPMQQTDLEAIVGIIA

QRACDQLDTIVVEDCLNGEATDSCQCDEGYGGARCETKCMNGDVVSTSPYNGQICDCELGFGGSDCSMESDSYHFMAEAQ

TGALRCQALFNDHTGYLYDEPGA

>SB4_SEC_allele_2

KSNVPLLSPLDSGEFEGSEYAVRTGWPYIHIAHFNNSDRSRESVSAGIKATPSGLTFSTPINTTGTIDSCTYSAYTVLSV

KEEATSSVEFVQFAVFLTDADPQFGRKIVYNGRVHGSKGRSTISVKGNPNNVVVGSVVLTKTGADPMQQTDLEAIVRIIA

QRACDQLDTIVVEDCLNGELTDTCQCDEGYGGARCETKCMNGDVVSTSPYNGQICNCELGFGGSDCSMESDSYHFMARAQ

TGALKCQALFNDHTGYLYDEPGA

>SB6_SEC_allele_1

KSNVPLLSPSDSGEFEGSEYAVRTGWPYIHIAYFNNSDRSRESVSAGIKASSSGLTFSTPINTTGTVDSCTYSAHTLLSV

KDEATSSVEFVQFTVYLTEADPQVTKIIYNSRVHGSIGRSTISVKGNPNNVVVGSVVLSKTGADPMQQTDLEAIVRIIAQ

RACDRLDTIVVEDCLNGEVTDSCQCDEGYGGARCETKCMNGDVVSTSPYNGQICDCELGFGGSDCSKESDSYHFMAKAQT

GALKCQALFNDHTGYLYDEPGA

>SB6_SEC_allele_2

KSNVPLLSPSDSGEFEGSEYAVRTGWPYIHIAYFNNSDRSRESVSAGIKTTPSGLTFSTPINTTGTIDSCTYSAYTVLSL

KEDETSSVEFVQFAVYLTDPQFGQKIVYNGRVRGSKGRSTISVKGNPNNVVVGSVVVTKTGADPMQQTDLESIVGIIAQR

ACDQLDTIVVEDCLNGDATDSCQCDEGYGGARCETKCMNGDVVSTSPYNGQICDCELGFGGSDCSMESDSYHFMAKAQTG

ALKCQALFNDHTGYLYDEPGA

**fuhc transmembrane cDNA sequences**

>32e_TM_allele_1

CAAGATCCTACAGGAAGTATCAGCGCGATCATGAAGAAAGCATTTATTTTGCAAGTAATTCTGGGAGTGTTGAGGCTTT

CCGACAGTGTCGACTTGAAGTACGACGATGTTTCGTACGCTACTGTACACCAAAGCGCTTTCCTTCGATGCGGGATGAT

GCCGGTGCAGCCTATAAAGGAACTTTGGTGGTTCAAAATTGGCGCCTCGGAAGAGCGAGTCTACTATTACGATCCTACA

TCAGTTGATGCAGCTCTGATGAAAAGAGCGAGACGGTCTAGCCGTGACAACACATCCCTCGAAATACATCCTACTCAAT

TGAGCGACGAGGGAGAGTACAAATGCCGTATCGTGGTCTGGGACAACACAGGAAATTCTACCGAGCCATCAGTGCCAAT

CTCGAAAGAAGCAAGAATGATGCTGAAAGTGAACGTTCCCCCGGAAAAAGTGGAAATAACGCACCTCACGAGCGGTAGA

AATTTGTCTTTGAGATGTAAATCCGAAGGAGCAAAGCCGCCGGCTGATATCCTATGGACCAAAACGTCACCTGACGGAG

TGGAGACTGAAATACCTGATTTATCCAAGACAAGGGAGATAGAGCCTGCAGGAGAGTTATATGACACGGCCGAACAAAT

CTTCTACCAGTTGGAAGAAAATGAAATGATCGCCAAAATATCGTGCGTCGTCAGGCATCACAACACAAACACAACAATT

TATAAGAATATAACATTCGTTTCTCCCGCTATACAGAACGCGCTCAAAGTCACAATACAAAGATTTGCTGAACCAGTCT

TATATTCTTCCATTGAAAATCAAGATGGTCAAGATAATAAGGCAGGCTTGGTGAAAATTTCTGCCGATCCCGGATTCAA

AGTACCCTACAATACTTCGGTAACTCTTAAAGCTGCTGCTCCTGGAGTATTTCCCGAGCCTAGGTATTTGTGGTGGGTT

GGTGACCAGACAGGAATAGCCGGACGAGAATTGCGCATCGACAATTTAGAAGAAGACAAGTCTTATTATGTACTCGCGG

AGAACCCATTGGGGCGTGTGGTACTTGAGGTACCGGTTCATATCACCACTTCAGATTATAGCCAATGGAACGACAGCTC

TAGAGACGGTGATGTGAATGAAGGCGACACAACCGAAGGCTTGGACACAGGAGCAATCATAGGAATAGTGATCGGAGCG

CTGATTGCAGTTTTCTTCATTGGATTCGCGGTGTGGTATTGGACCATCAGAGATGGAACACAAGTATTCTCTCCAAAAA

AGGAAAAACCACATACCATTGATGAAGAGACTGTACCATTCAGATCTACCGAGAAGCCTCTTCTGAGGACAATTTCTAC

ACAGACATTGGAGGGTCGAAGATCAAGGCGAGAGCCAAGTATC

>32e_TM_allele_2

CAAGATCCTACAGGAAGTATCAGCGCGATCATGAAGAAAGCATTTATTTTGCAAGTAATTCTGGGAGTGTTGAGGCTTT

CCGACAGTGTCGACTTGAAGTACGACGATGTTTCGTACGCTACTGTACACCAAAGCGCTTTCCTTCGATGCGGGATGAT

GCCTGTGCAGCCTATAAAGGAACTTTGGTGGTTCAAAATTGGCGCCTCGGAAGAGCGAGTCTACTATTACGATCCTACA

TCAGTTGATGCAGCTCTGATGAAAAGAGCGAGACGATCTAACCGTGACAACACATCCCTCGAAATATATCCTACACAAT

TGAGCGACGAGGGGGAGTACAAATGCCGTATCGTGGTATGGGACAACACAGGAAATTCTACCGAGCCATCAGTGCCAAT

CTCGAAAGAAGCAAGAATGATGCTGAAAGTTAACGTTCCCCCGGAAAAAGTGGAAATAACGCACCTCACGAGCGGTAGA

AATTTGTCTTTGAGATGTAAATCCGAAGGAGCAAAGCCGCCGGCTGATATCCTATGGACTAAAACGTCACCTGACGGAG

TGGAGACTGAAATACCCGATTTAGCCAAGACAAGGGAGATCGAGCCTGCAGGAGAGTTATATGACACAGCCGAACAGAT

CTTCTACCAGTTGGAAGAAAATGAAATGATCGCCAAAATATCGTGCGTCGTCAGGCACCACAACACAAACACAACAATT

TATAAAAACATAACATTCGTTTCTCCCGCTATACAGAACGCGCTCAAAGTCACAATACAAAGATTTGCCGAACCAGTCT

TATATTCTTCCATTGAAAATCAAGATGGTCAAGATAATAAGGCAGGCTTGGTGAAAATTTCTGCCGATCCCGGATTCAA

AGTACCCTACAATACTTCGGTAACTCTTAAAGCTGCTGCTCCTGGAGTATTTCCCGAGCCTAGGTATTTGTGGTGGGTT

GGTGACCAGACTGGAATAGCCGGACGAGAATTGCGCATCGACAATTTAGAAGAAGACAAGTCTTATTATGTACTCGCGG

AGAACCCATTGGGGCGTGTGGTACTTGAGGTACCGGTTCATATCACCATTTCAGATTATAGCCAATGGAACGACAGCTC

TAGAGACGGTGATGTGAATGAAGGCGACACAACCGAAGGCTTGGACACAGGAGCAATCATAGGAATAGTAATCGGAGCG

CTGATTGCAGTTTTCTTCATTGGATTCGCGGTGTGGTATTGGACCATCAGAGATGGAACACAAGTATTCTCTCCAAAAA

AGGAAAAACCACATACCATTGATGAAGAGACTGTACCATTCAGATCTACCGAGAAGCCTCTTCTGAGGACAATTTCTAC

ACAGACATTGGAGGGTCGAAGATCAAGGCGAGAGCCAAGTATC

>HM9AY x 1225F1_TM_allele_1

CAAGATCCTACAGGAAGTATCAGCGCGATCATGAAGAAAGCATTTATTTTGCAAGTAATTCTGGGAGTGTTGAGGCTTT

CGGACAGTGTCGACTTGGAGTACGACGATGTTTCGTACGCTACTGTACACCAAAGCGCTTTCCTTCGATGCGGGATGAT

GCCGGTGCAGCCTATAAAGGAACTTTGGTGGTTCAAAATTGGCGCCTCGGAAGAGCGAGTCTACTATTACGATCCTACA

TCAGTTGATGCAGCTCTGATGAAAAGAGCGAGACGGTCTAGCCGTGACAACACATCCCTCGAAATATGTCCTACACAAT

TGAGCGACGAGGGGGAGTACAAATGCCGTATCGTGGTTTGGGACAACACAGGAGATTCTACCGAGCCATCAGTGCCAAT

CTCGAAAGAAGCAAGAATGATGCTGAAAGTGAACGTTCCCCCGGAAAAAGTGGAAATAACGCACCTCACGAGCGGTAGA

AATTTGTCTTTGAGATGTAAAGCCGAAGGAGCAAAGCCGCCGGCTGATATCCTATGGACTAAAACGTCACCTGACGGAG

TGGAGACTGAAATACCTGATTTATCCAAGACAAGGGAGATCGAGCCTGCAGGAGAGTTATATGACACAGCCGAACAGAT

CTTCTACCAGTTGGAAGAAAATGAAATGATCGCCAAAATATCGTGTGTCGTCAGGCACCACAACACAAACACAACAATT

TATAAAAATATAACATTCGTTTCTCCCGCTATACAGAACGCGCTCAAAGTCACAATACAAAGATTTGCCGAACCAGTCT

TATATTCTTCCATCGAAAATCAAGATGGTCAAGATAATAAGGCAGGCCTGGTGAAAATTTCAGCCGATCCCGGATTCAA

AGTACCCTACAATACTTCGGTAACTCTTAAAGCTTCTGCTCCTGGAGTATTTCCCGAGCCGAGGTATTTGTGGTGGGTT

GGTGACCAGACAGGAATAGCTGGACGAGAATTGCACATCGACAATTTAGAAGAAGACAAGTCTTATTATGTACTCGCGG

AGAACCCATTGGGGCGTGTGGTACTTGAGGTACCTGTCCATATCACCACTTCAGATTATAGCCAATGGAACGACAGCTC

TAGAGACGGTGATGTGATTGTTGACGACACAACCGGAGGCTTGGACACAGGAGCAATCATAGGAATAGTGATCGGAGCG

CTGATCGCAGTTTTCTTCATTGGATTCGCGGTGTGGTATTGGACCATCAGAGATGGAACGCAAGTCTTCTCTCCGAAAA

AGGAAAAACCACATACCATTGATGAGGAGACTATACCATTCAGATCCACGGACAAGCCTCTTCTGAGGACAATTTCTAC

GCAGACATTGGAGGGTCGAAGATCAAGGCGAGAGCCAAGTATC

>HM9AY x 1225F1_TM_allele_2

CAAGATCCTACAGGAAGTATCAGCGCGATCATGAAGAAAGCATTTATTTTGCAAGTAATTCTGGGAGTGTTGAGGCTTT

CCGACAGTGTCGACTTGAAGTACGACGATGTTTCGTACGCTACTGTACACCAAAGCGCTTTCCTTCGATGCGGGATGAT

GCCTGTGCAGCCTATAAAGGAACTTTGGTGGTTCAAAATTGGCGCCTCGGAAGAGCGAGTCTACTATTACGATCCTACA

TCAGTTGATGCAGCTCTGATGAAAAGAGCGAGACGATCTAACCGTGACAACACATCCCTCGAAATATATCCTACACAAT

TGAGCGACGAGGGGGAGTACAAATGCCGTATCGTGGTATGGGACAACACAGGAAATTCTACCGAGCCATCAGTGCCAAT

CTCGAAAGAAGCAAGAATGATGCTGAAAGTTAACGTTCCCCCGGAAAAAGTGGAAATAACGCACCTCACGAGCGGTAGA

AATTTGTCTTTGAGATGTAAATCCGAAGGAGCAAAGCCGCCGGCTGATATCCTATGGACTAAAACGTCACCTGACGGAG

TGGAGACTGAAATACCCGATTTAGCCAAGACAAGGGAGATCGAGCCTGCAGGAGAGTTATATGACACAGCCGAACAGAT

CTTCTACCAGTTGGAAGAAAATGAAATGATCGCCAAAATATCGTGCGTCGTCAGGCACCACAACACAAACACAACAATT

TATAAAAACATAACATTCGTTTCTCCCGCTATACAGAACGCGCTCAAAGTCACAATACAAAGATTTGCCGAACCAGTCT

TATATTCTTCCATTGAAAATCAAGATGGTCAAGATAATAAGGCAGGCTTGGTGAAAATTTCTGCCGATCCCGGATTCAA

AGTACCCTACAATACTTCGGTAACTCTTAAAGCTGCTGCTCCTGGAGTATTTCCCGAGCCTAGGTATTTGTGGTGGGTT

GGTGACCAGACTGGAATAGCCGGACGAGAATTGCGCATCGACAATTTAGAAGAAGACAAGTCTTATTATGTACTCGCGG

AGAACCCATTGGGGCGTGTGGTACTTGAGGTACCGGTTCATATCACCATTTCAGATTATAGCCAATGGAACGACAGCTC

TAGAGACGGTGATGTGAATGAAGGCGACACAACCGAAGGCTTGGACACAGGAGCAATCATAGGAATAGTAATCGGAGCG

CTGATTGCAGTTTTCTTCATTGGATTCGCGGTGTGGTATTGGACCATCAGAGATGGAACACAAGTATTCTCTCCAAAAA

AGGAAAAACCACATACCATTGATGAAGAGACTGTACCATTCAGATCTACCGAGAAGCCTCTTCTGAGGACAATTTCTAC

ACAGACATTGGAGGGTCGAAGATCAAGGCGAGAGCCAAGTATC

>SC27f_TM_allele_1

CAAGATCCTACAGGAAGTATCAGCGCGATCATGAAGAAAGCATTTATTTTGCAAGTAATTCTGGGAGTGTTGAGGCTTT

CGGACAGTGTCGACTTGGAGTACGACGATGTTTCGTACGCTACTGTACACCAAAGCGCTTTCCTTCGATGCGGGATGAT

GCCGGTGCAGCCTATAAAGGAACTTTGGTGGTTCAAAATTGGCGCCTCGGAAGAGCGAGTCTACTATTACGATCCTACA

TCAGTTGATGCAGCTCTGATGAAAAGAGCGAGACGGTCTAGCCGTGACAACACATCCCTCGAAATATGTCCTACACAAT

TGAGCGACGAGGGGGAGTACAAATGCCGTATCGTGGTTTGGGACAACACAGGAGATTCTACCGAGCCATCAGTGCCAAT

CTCGAAAGAAGCAAGAATGATGCTGAAAGTGAACGTTCCCCCGGAAAAAGTGGAAATAACGCACCTCACGAGCGGTAGA

AATTTGTCTTTGAGATGTAAAGCCGAAGGAGCAAAGCCGCCGGCTGATATCCTATGGACTAAAACGTCACCTGACGGAG

TGGAGACTGAAATACCTGATTTATCCAAGACAAGGGAGATCGAGCCTGCAGGAGAGTTATATGACACAGCCGAACAGAT

CTTCTACCAGTTGGAAGAAAATGAAATGATCGCCAAAATATCGTGTGTCGTCAGGCACCACAACACAAACACAACAATT

TATAAAAATATAACATTCGTTTCTCCCGCTATACAGAACGCGCTCAAAGTCACAATACAAAGATTTGCCGAACCAGTCT

TATATTCTTCCATCGAAAATCAAGATGGTCAAGATAATAAGGCAGGCCTGGTGAAAATTTCAGCCGATCCCGGATTCAA

AGTACCCTACAATACTTCGGTAACTCTTAAAGCTTCTGCTCCTGGAGTATTTCCCGAGCCGAGGTATTTGTGGTGGGTT

GGTGACCAGACAGGAATAGCTGGACGAGAATTGCACATCGACAATTTAGAAGAAGACAAGTCTTATTATGTACTCGCGG

AGAACCCATTGGGGCGTGTGGTACTTGAGGTACCTGTCCATATCACCACTTCAGATTATAGCCAATGGAACGACAGCTC

TAGAGACGGTGATGTGATTGTTGACGACACAACCGGAGGCTTGGACACAGGAGCAATCATAGGAATAGTGATCGGAGCG

CTGATCGCAGTTTTCTTCATTGGATTCGCGGTGTGGTATTGGACCATCAGAGATGGAACGCAAGTCTTCTCTCCGAAAA

AGGAAAAACCACATACCATTGATGAGGAGACTATACCATTCAGATCCACGGACAAGCCTCTTCTGAGGACAATTTCTAC

GCAGACATTGGAGGGTCGAAGATCAAGGCGAGAGCCAAGTATC

>SC27f_TM_allele_2

CAAGATCCTACAGGAAGTATCAGCGCGATCATGAAGAAAGCATTTATTTTGCAAGTAATTCTGGGAGTGTTGAGGCTTT

CGGACAGTGTCGACTTGGAGTACGACGATGTTTCGTACGCTACTGTACACCAAAGCGCTTTCCTTCGATGCGGGATGAT

GCCGGTGCAGCCTATAAAGGAACTTTGGTGGTTCAAAATTGGCGCCTCGGAAGAGCGAGTCTACTATTACGATCCTACA

TCAGTTGATGCAGCTCTGATGAAAAGAGCGAGACGGTCTAGCCGTGACAACACATCCCTCGAAATATGTCCTACACAAT

TGAGCGACGAGGGGGAGTACAAATGCCGTATCGTGGTTTGGGACAACACAGGAGATTCTACCGAGCCATCAGTGCCAAT

CTCGAAAGAAGCAAGAATGATGCTGAAAGTGAACGTTCCCCCGGAAAAAGTGGAAATAACGCACCTCACGAGCGGTAGA

AATTTGTCTTTGAGATGTAAAGCCGAAGGAGCAAAGCCGCCGGCTGATATCCTATGGACTAAAACGTCACCTGACGGAG

TGGAGACTGAAATACCTGATTTATCCAAGACAAGGGAGATCGAGCCTGCAGGAGAGTTATATGACACAGCCGAACAGAT

CTTCTACCAGTTGGAAGAAAATGAAATGATCGCCAAAATATCGTGTGTCGTCAGGCACCACAACACAAACACAACAATT

TATAAAAATATAACATTCGTTTCTCCCGCTATACAGAACGCGCTCAAAGTCACAATACAAAGATTTGCCGAACCAGTCT

TATATTCTTCCATCGAAAATCAAGATGGTCAAGATAATAAGGCAGGCCTGGTGAAAATTTCAGCCGATCCCGGATTCAA

AGTACCCTACAATACTTCGGTAACTCTTAAAGCTTCTGCTCCTGGAGTATTTCCCGAGCCGAGGTATTTGTGGTGGGTT

GGTGACCAGACAGGAATAGCTGGACGAGAATTGCACATCGACAATTTAGAAGAAGACAAGTCTTATTATGTACTCGCGG

AGAACCCATTGGGGCGTGTGGTACTTGAGGTACCTGTCCATATCACCACTTCAGATTATAGCCAATGGAACGACAGCTC

TAGAGACGGTGACGCGATGGTTAACGACACAACCGAAGGCTTGGACACAGGGGCAATCATAGGAATAGTGATCGGAGCA

CTGATCGCAGTTTTCTTCATTGGATTCGCGGTGTGGTATTGGACCATCAGAGATGGAACGCATGTCTTCTCTCCCAAAA

AGGAAAAACCGCATACCATTGATGAGGAGACTATACCATTCAGATCCACGGACAAGCCTCTTCTGAGGACAATTTCTAC

GCAGACATTGGAGGGTCGGAGATCAAGGCGAGAGCCAAGTATC

>SB1_TM_allele_1

CAAGATCCTACAGGAAGTATCAGCGCGATCATGAAGAAAGCATTTATTTTGCAAGTAATTCTGGGAGTGTTGAGGCTTT

CCGACAGTGTCGACTTGAAGTACGACGATGTTTCGTACGCTACTGTACACCAAAGCGCTTTCCTTCGATGCGGGATGAT

GCCGGTGCAGCCTATAAAGGAACTTTGGTGGTTCAAAATTGGCGCCTCGGAAGAGCGAGTCTACTATTACGATCCTACA

TCAGTTGATGCAGCTCTGATGAAAAGAGCGAGACGGTCTAGCCCTGACAACACATCCCTCGAAATACATCCTACTCAAT

TGAGCGACGAGGGAGAGTACAAATGCCGTATCGTGGTCTGGGACAACACAGGAAATTCTACCGAGCCATCAGTGCCAAT

CTCGAAAGAAGCAAGAATGATGCTGAAAGTGAACGTTCCCCCGGAAAAAGTGGAAATAACGCACCTCACGAGCGGTAGA

AATTTGTCTTTGAGATGTAAATCCGAAGGAGCAAAGCCGCCGGCTGATATCCTATGGACCAAAACGTCACCTGACGGAG

TGGAGACTGAAATACCTGATTTATCCAAGACAAGGGAGATAGAGCCTGCAGGAGAGTTATATGATACGGCCGAACAAAT

CTTCTACCAGTTGGAAGAAAATGAAATGATCGCCAAAATATCGTGCGTCGTCAGGCATCACAACACAAACACAACAATT

TATAAGAATATAACATTCGTTTCTCCCGCTATACAGAACGCGCTCAAAGTCACAATACAAAGATTTGCTGAACCAGTCT

TATATTCTTCCATTGAAAATCAAGATGGTCAAGATAATAAGGCAGGCTTGGTGAAAATTTCTGCCGATCCCGGATTCAA

AGTACCCTACAATACTTCGGTAACTCTTAAAGCTGCTGCTCCTGGAGTATTTCCCGAGCCTAGGTATTTGTGGTGGGTT

GGTGACCAGACAGGAATAGCCGGACGAGAATTGCGCATCGACAATTTAGAAGAAGACAAGTCTTATTATGTACTCGCGG

AGAACCCATTGGGGCGTGTGGTACTTGAGGTAC

>SB1_TM_allele_2

CAAGATCCTACAGGAAGTATCAGCGCGATCATGAAGAAAGCATTTATTTTGCAAGTAATTCTGGGAGTGTTGAGGCTTTC

CGACAGTGTTGACTTGAAGTACGACGATGTTTCGTACGCTACTGTACACCAAAGCGCTTTCCTTCGATGCGGGATGATGC

CGGTGCAGCCTATAAAGGAACTTTGGTGGTTCAAAATTGGCGCCTCGGAAGAGCGAGTCTACTATTACGATCCTACATCA

GTTGATGCAGCTCTGATGAAAAGAGCGAGACGATCTAACCGTGACAACACATCCCTCGAAATATATCCTACACAATTGAG

CGACGAGGGGGAGTACAAATGCCGTATCGTGGTATGGGACAACACAGGAAATTCTACCGAGCCATCAGTGCCAATCTCGA

AAGAAGCAAGAATGATGCTGAAAGTTAACGTTCCCCCGGAAAAAGTGGAAATAACGCACCTCACGAGCGGTAGAAATTTG

TCTTTGAGATGTAAATCCGAAGGAGCAAAGCCGCCGGCTGATATCCTATGGACTAAAACGTCACCTGACGGAGTGGAGAC

TGAAATACCCGATTTAGCCAAGACAAGGGAGATCGAGCCTGCAGGAGAGTTATATGACACAGCCGAACAGATCTTCTACC

AGTTGGAAGAAAATGAAATGATCGCCAAAATATCGTGCGTCGTCAGGCACCACAACACAAACACAACAATTTATAAAAAT

ATAACATTCGTTTCTCCCGCTATACAGAACGCGCTCAAAGTCACAATACAAAGATTTGCCGAACCAGTCTTATATTCTTC

CATCGAAAATCAAGATGGTCGAGATAATAAGGCAGGCCTGGTGAAAATTTCAGCCGATCCCGGATTCAAAGTACCCTACA

ATACTTCGGTAACTCTTAAAGCTGCTGCTCCTGGAGTATTTCCCGAGCCGAGGTATTTGTGGTGGGTTGGTGACCAGACA

GGAATAGCCGGACGAGAATTGCACATAGACAATTTGGAAGAAGACAAGTCTTATTATGTACTCGCGGAGAACCCATTGGG

GCGTGTGGTACTTGAGGTAC

>SB2_TM_allele_1

CAAGATCCTACAGGAAGTATCAGCGCGATCATGAAGAAAGCATTTATTTTGCAAGTAATTCTGGGAGTGTTGAGGCTTTC

GGACAGTGTCGACTTGGAGTACGACGATGTTTCGTACGCTACTGTACACCAAAGCGCTTTCCTTCGATGCGGGATGATGC

CGGTGCAGCCTATAAAGGAACTTTGGTGGTTCAAAATTGGCGCCTCGGAAGAGCGAGTCTACTATTACGATCCTACATCA

GTTGATGCAGCTCTGATGAAAAGAGCGAGACGGTCTAGCCGTGACAACACATCCCTCGAAATATGTCCTACACAATTGAG

CGACGAGGGGGAGTACAAATGCCGTATCGTGGTTTGGGACAACACAGGAGATTCTACCGAGCCATCAGTGCCAATCTCGA

AAGAAGCAAGAATGATGCTGAAAGTGAACGTTCCCCCGGAAAAAGTGGAAATAACGCACCTCACGAGCGGTAGAAATTTG

TCTTTGAGATGTAAATCTGAAGGAGCAAAGCCGCCGGCTGATATCCTATGGACTAAAACGTCACCTGACGGAGTGGAGAC

TGAAATACCTGATTTATCCAAGACAAGGGAGATAGAGCCTGCAGGAGAGTTATATGACACGGCCGAACAGATCTTCTACC

AGTTGCAAGAAAATGAAATGATCGCCAAAATATCGTGCGTCGTCAGGCACCACAACACAAACACAACAATTTATAAAAAT

ATAACATTCGTTTCTCCCGCTATACAGAACGCGCTCAAAGTCACAATACAAAGATTTGCCGAACCAGTCTTATATTCTTC

CATCGAAAATCAAGATGGTCAAGATAATAAGGCAGGCCTGGTGAAAATTTCAGCCGATCCCGGATTCAAAGTACCCTACA

ATACTTCGGTAACTCTTAAAGCTTCTGCTCCTGGAATATTTCCCGAGCCGAGGTATTTGTGGTGGGTTGGTGACCAGACA

GGAATAGCCGGACGAGAATTGCACATCGACAATTTGGAAGAAGACAAGTCTTATTATGTACTCGCGGAGAACCCATTGGG

GCGTGTGGTACTTGAGGTAC

>SB2_TM_allele_2

CAAGATCCTACAGGAAGTATCAGCGCGATCATGAAGAAAGCATTTATTTTGCAAGTAATTCTGGGAGTGTTGAGGCTTT

CCGACAGTGTCGACTTGAAGTACGACGATGTTTCGTACGCTACTGTACACCAAAGCGCTTTCCTTCGATGCGGGATGAT

GCCTGTGCAGCCTATAAAGGAACTTTGGTGGTTCAAAATTGGCGCCTCGGAAGAGCGAGTCTACTATTACGATCCTACA

TCAGTTGATGCAGCTCTGATGAAAAGAGCGAGACGGTCTAGCCGTGACAACACATCCCTCGAAATATATCCTACACAAT

TGAGCGACGAGGGGGAGTACAAATGCCGTATCGTGGTATGGGACAACACAGGAAATTCTACCGAGCCATCAGTGCCAAT

CTCGAAAGAAGCAAGAATGATGCTGAAAGTGAACGTACCCCCGGAAAAAGTGGAAATAACGCACCTCACGAGCGGTAGA

AATTTGTCTTTGAGATGTAAATCTGAAGGAGCAAAGCCGCCGGCTGATATCCTATGgaccaaaacgtcacctgaCGGAG

TGGAGACTGAAATACCTGATTTATcCAAGACAAGGGAGATAGAGCCTGCAGGAGAGTTATATGACACGGCCGAACAGAT

CTTCTACCAGTTGCAAGAAAATGAAATGATCGCCAAAATATCGTGCGTCGTCAGGCACCACAACACAAACACAACAATT

TATAAAAATATAACATTCGTTTCTCCCGCTATACAGAACGCGCTCAAAGTCACAATACAAAGATTTGCCGAACCAGTCT

TATATTCTTCCATCGAAAATCAAGATGGTCAAGATAATAAGGCAGGCCTGGTGAAAATTTCAGCCGATCCCGGATTCAA

AGTACCCTACAATACTTCGGTAACTCTTAAAGCTTCTGCTCCTGGAATATTTCCCGAGCCGAGGTATTTGTGGTGGGTT

GGTGACCAGACAGGAATAGCCGGACGAGAATTGCACATCGACAATTTGGAAGAAGACAAGTCTTATTATGTACTCGCGG

AGAACCCATTGGGGCGTGTGGTACTTGAGGTAC

>SB3_TM_allele_1

CAAGATCCTACAGGAAGTATCAGCGCGATCATGAAGAAAGCATTTATTTTGCAAGTAATTCTGGGAGTGTTGAGGCTTTC

GGACAGTGTCGACTTGGAGTACGACGATGTTTCGTACGCTACTGTACACCAAAGCGCTTTCCTTCGATGCGGGATGATGC

CGGTGCAGCCTATAAAGGAACTTTGGTGGTTCAAAATTGGCGCCTCGGAAGAGCGAGTCTACTATTACGATCCTACATCA

GTTGATGCAGCTCTGATGAAAAGAGCGAGACGGTCTAGCCGTGACAACACATCCCTCGAAATATGTCCTACACAATTGAG

CGACGAGGGGGAGTACAAATGCCGTATCGTGGTTTGGGACAACACAGGAGATTCTACCGAGCCATCAGTGCCAATCTCGA

AAGAAGCAAGAATGATGCTGAAAGTGAACGTTCCCCCGGAAAAAGTGGAAATAACGCACCTCACGAGCGGTAGAAATTTG

TCTTTGAGATGTAAATCTGAAGGAGCAAAGCCGCCGGCTGATATCCTATGGACTAAAACGTCACCTGACGGAGTGGAGAC

TGAAATACCTGATTTATCCAAGACAAGGGAGATAGAGCCTGCAGGAGAGTTATATGACACGGCCGAACAGATCTTCTACC

AGTTGCAAGAAAATGAAATGATCGCCAAAATATCGTGCGTCGTCAGGCACCACAACACAAACACAACAATTTATAAAAAT

ATAACATTCGTTTCTCCCGCTATACAGAACGCGCTCAAAGTCACAATACAAAGATTTGCCGAACCAGTCTTATATTCTTC

CATCGAAAATCAAGATGGTCAAGATAATAAGGCAGGCCTGGTGAAAATTTCAGCCGATCCCGGATTCAAAGTACCCTACA

ATACTTCGGTAACTCTTAAAGCTTCTGCTCCTGGAATATTTCCCGAGCCGAGGTATTTGTGGTGGGTTGGTGACCAGACA

GGAATAGCCGGACGAGAATTGCACATCGACAATTTGGAAGAAGACAAGTCTTATTATGTACTCGCGGAGAACCCATTGGG

GCGTGTGGTACTTGAGGTAC

>SB3_TM_allele_2

CAAGATCCTACAGGAAGTATCAGCGCGATCATGAAGAAAGCATTTATTTTGCAAGTAATTCTGGGAGTGTTGAGGCTTT

CCGACAGTGTTGACTTGAAGTACGACGATGTTTCGTACGCTACTGTACACCAAAGCGCTTTCCTTCGATGCGGGATGAT

GCCGGTGCAGCCTATAAAGGAACTTTGGTGGTTCAAAATTGGCGCCTCGGAAGAGCGAGTCTACTATTACGATCCTACA

TCAGTTGATGCAGCTCTGATGAAAAGAGCGAAGCGGTCTAGCCGTGACAACACATCACTCGAAATACATCCTACTCATT

TGAGCGACGAGGTAGAGTACAAATGCCGTATCGTGGTCTGGGACAACACAGGAAATTCTACCGAGCCATCAGTGCCAAT

CTCGAAAGAAGCAAGAATGATGCTGAAAGTGAATGTTCCCCCGGAAAAAGTGGAAATAACGCACCTCACGAGCGGTAGA

AATTTGTCTTTGAGATGTAAATCCGAAGGAGCAAAGCCGCCGGCTGATATCCTATGGACTAAAACGTCACCTGACGGAG

TGGAGACTGAAATACCTGATTTATCCAAGACAAGGGAGATAGAGCCTGCAGGAGAGTTATATGACACGGCCGAACAGAT

CTTCTACCAGTTGGAAGAAAATGAAATGATCGCCAAAATATCGTGTGTCGTCAGGCACCACAACACAAACACAACAATT

TATAAAAATATAACGTTCGTTTCTCCGGCTATACAGAACGCGCTCAAAGTCACAATACAAAGATTTGCCGAACCAGTCT

TATATTCTTCCATTGAAAATCAAGGTGGTCAAGATAGTAAGGCAGGTCTGGTGAAAATTTCAGCTGATCCCGGATTCAA

AGTACCCTACAATACTTCGGTAACTCTTAAAGCTTCTGCTCCTGGAGTATTTCCCGAGCCAAGGTATTTGTGGTGGGTT

GGTGACCAGACAGGAATAGCCGGACGAGAATTGCGCATCGACAATTTGGAAGAAGACAGGTCTTATTATGTACTCGCGG

AGAACCCATTGGGGCGTGTGGTACTTGAGGTAC

>SB4_TM_allele_1

CAAGATCCTACAGGAAGTATCAGCGCGATCATGAAGAAAGCATTTATTTTGCAAGTAATTCTGGGAGTGTTGAGGCTTT

CCGACAGTGTCGACTTGAAGTACGACGATGTTTCGTACGCTACTGTACACCAAAGCGCTTTCCTTCGATGCGGGATGAT

GCCTGTGCAGCCTATAAAGGAACTTTGGTGGTTCAAAATTGGCGCCTCGGAAGAGCGAGTCTACTATTACGATCCTACA

TCAGTTGATGCAGCTCTGATGAAAAGAGCGAGACGGTCTAGCCGTGACAACACATCCCTCGAAATATATCCTACACAAT

TGAGCGACGAGGGGGAGTACAAATGCCGTATCGTGGTATGGGACAACACAGGAAATTCTACCGAGCCATCAGTGCCAAT

CTCGAAAGAAGCAAGAATGATGCTGAAAGTGAACGTACCCCCGGAAAAAGTGGAAATAACGCACCTCACGAGCGGTAGA

AATTTGTCTTTGAGATGTAAATCTGAAGGAGCAAAGCCGCCGGCTGATATCCTATGgaccaaaacgtcacctgaCGGAG

TGGAGACTGAAATACCTGATTTATcCAAGACAAGGGAGATAGAGCCTGCAGGAGAGTTATATGACACGGCCGAACAGAT

CTTCTACCAGTTGCAAGAAAATGAAATGATCGCCAAAATATCGTGCGTCGTCAGGCACCACAACACAAACACAACAATT

TATAAAAATATAACATTCGTTTCTCCCGCTATACAGAACGCGCTCAAAGTCACAATACAAAGATTTGCCGAACCAGTCT

TATATTCTTCCATCGAAAATCAAGATGGTCAAGATAATAAGGCAGGCCTGGTGAAAATTTCAGCCGATCCCGGATTCAA

AGTACCCTACAATACTTCGGTAACTCTTAAAGCTTCTGCTCCTGGAATATTTCCCGAGCCGAGGTATTTGTGGTGGGTT

GGTGACCAGACAGGAATAGCCGGACGAGAATTGCACATCGACAATTTGGAAGAAGACAAGTCTTATTATGTACTCGCGG

AGAACCCATTGGGGCGTGTGGTACTTGAGGTAC

>SB4_TM_allele_2

CAAGATCCTACAGGAAGTATCAGCGCGATCATGAAGAAAGCATTTATTTTGCAAGTAATTCTGGGAGTGTTGAGGCTTT

CCGACAGTGTTGACTTGAAGTACGACGATGTTTCGTACGCTACTGTACACCAAAGCGCTTTCCTTCGATGCGGGATGAT

GCCGGTGCAGCCTATAAAGGAACTTTGGTGGTTCAAAATTGGCGCCTCGGAAGAGCGAGTCTACTATTACGATCCTACA

TCAGTTGATGCAGCTCTGATGAAAAGAGCGAAGCGGTCTAGCCGTGACAACACATCACTCGAAATACATCCTACTCATT

TGAGCGACGAGGTAGAGTACAAATGCCGTATCGTGGTCTGGGACAACACAGGAAATTCTACCGAGCCATCAGTGCCAAT

CTCGAAAGAAGCAAGAATGATGCTGAAAGTGAATGTTCCCCCGGAAAAAGTGGAAATAACGCACCTCACGAGCGGTAGA

AATTTGTCTTTGAGATGTAAATCCGAAGGAGCAAAGCCGCCGGCTGATATCCTATGGACTAAAACGTCACCTGACGGAG

TGGAGACTGAAATACCTGATTTATCCAAGACAAGGGAGATAGAGCCTGCAGGAGAGTTATATGACACGGCCGAACAGAT

CTTCTACCAGTTGGAAGAAAATGAAATGATCGCCAAAATATCGTGTGTCGTCAGGCACCACAACACAAACACAACAATT

TATAAAAATATAACGTTCGTTTCTCCGGCTATACAGAACGCGCTCAAAGTCACAATACAAAGATTTGCCGAACCAGTCT

TATATTCTTCCATTGAAAATCAAGGTGGTCAAGATAGTAAGGCAGGTCTGGTGAAAATTTCAGCTGATCCCGGATTCAA

AGTACCCTACAATACTTCGGTAACTCTTAAAGCTTCTGCTCCTGGAGTATTTCCCGAGCCAAGGTATTTGTGGTGGGTT

GGTGACCAGACAGGAATAGCCGGACGAGAATTGCGCATCGACAATTTGGAAGAAGACAGGTCTTATTATGTACTCGCGG

AGAACCCATTGGGGCGTGTGGTACTTGAGGTAC

>SB6_TM_allele_1

CAAGATCCTACAGGAAGTATCAGCGCGATCATGAAGAAAGCATTTATTTTGCAAGTAATTCTGGGAGTGTTGAGGCTTT

CCGACAGTGTCGACTTGAAGTACGACGATGTTTCGTACGCTACTGTACACCAAAGCGCTTTCCTTCGATGCGGGATGAT

GCCGGTGCAGCCTATAAAGGAACTTTGGTGGTTCAAAATTGGCGCCTCGGAAGAGCGAGTCTACTATTACGATCCTACA

TCAGTTGATGCAGCTCTGATGAAAAGAGCGAGACGGTCTAGCCCTGACAACACATCCCTCGAAATACATCCTACTCAAT

TGAGCGACGAGGGAGAGTACAAATGCCGTATCGTGGTCTGGGACAACACAGGAAATTCTACCGAGCCATCAGTGCCAAT

CTCGAAAGAAGCAAGAATGATGCTGAAAGTGAACGTTCCCCCGGAAAAAGTGGAAATAACGCACCTCACGAGCGGTAGA

AATTTGTCTTTGAGATGTAAATCCGAAGGAGCAAAGCCGCCGGCTGATATCCTATGGACCAAAACGTCACCTGACGGAG

TGGAGACTGAAATACCTGATTTATCCAAGACAAGGGAGATAGAGCCTGCAGGAGAGTTATATGATACGGCCGAACAAAT

CTTCTACCAGTTGGAAGAAAATGAAATGATCGCCAAAATATCGTGCGTCGTCAGGCATCACAACACAAACACAACAATT

TATAAGAATATAACATTCGTTTCTCCCGCTATACAGAACGCGCTCAAAGTCACAATACAAAGATTTGCTGAACCAGTCT

TATATTCTTCCATTGAAAATCAAGATGGTCAAGATAATAAGGCAGGCTTGGTGAAAATTTCTGCCGATCCCGGATTCAA

AGTACCCTACAATACTTCGGTAACTCTTAAAGCTGCTGCTCCTGGAGTATTTCCCGAGCCTAGGTATTTGTGGTGGGTT

GGTGACCAGACAGGAATAGCCGGACGAGAATTGCGCATCGACAATTTAGAAGAAGACAAGTCTTATTATGTACTCGCGG

AGAACCCATTGGGGCGTGTGGTACTTGAGGTAC

>SB6_TM_allele_2

CAAGATCCTACAGGAAGTATCAGCGCGATCATGAAGAAAGCATTTATTTTGCAAGTAATTCTGGGAGTGTTGAGGCTTT

CCGACAGTGTCGACTTGAAGTACGACGATGTTTCGTACGCTACTGTACACCAAAGCGCTTTCCTTCGATGCGGGATGAT

GCCTGTGCAGCCTATAAAGGAACTTTGGTGGTTCAAAATTGGCGCCTCGGAAGAGCGAGTCTACTATTACGATCCTACA

TCAGTTGATGCAGCTCTGATGAAAAGAGCGAGACGATCTAACCGTGACAACACATCCCTCGAAATATATCCTACACAAT

TGAGCGACGAGGGGGAGTACAAATGCCGTATCGTGGTATGGGACAACACAGGAAATTCTACCGAGCCATCAGTGCCAAT

CTCGAAAGAAGCAAGAATGATGCTGAATGTTAACGTTCCCCCGGAAAAAGTGGAAATAACGCACCTCACGAGCGGTAGA

AATTTGTCTTTGAGATGTAAATCCGAAGGAGCAAAGCCGCCGGCTGATATCCTATGGACTAAAACGTCACCTGACGGAG

TGGAGACTGAAATACCTGATTTATCCAAGACAAGGGAGATAGAGCCTGCAGGAGAGTTATATGACACGGCCGAACAGAT

CTTCTACCAGTTGGAAGAAAATGAAATGATCGCCAAAATATCGTGTGTCGTCAGGCACCACAACACAAACACAACAATT

TATAAAAATATAACGTTCGTTTCTCCGGCTATACAGAACGCGCTCAAAGTCACAATACAAAGATTTGCCGAACCAGTCT

TATATTCTTCCATTGAAAATCAAGGTGGTCAAGATAGTAAGGCAGGTCTGGTGAAAATTTCAGCTGATCCCGGATTCAA

AGTACCCTACAATACTTCGGTAACTCTTAAAGCTTCTGCTCCTGGAGTATTTCCCGAGCCAAGGTATTTGTGGTGGGTT

GGTGACCAGACAGGAATAGCCGGACGAGAATTGCGCATCGACAATTTGGAAGAAGACAGGTCTTATTATGTACTCGCGG

AGAACCCATTGGGGCGTGTGGTACTTGAGGTAC

**fuhc transmembrane protein sequences**

>SC32e allele 1

MKKAFILQVILGVLRLSDSVDLKYDDVSYATVHQSAFLRCGMMPVQPIKELWWFKIGASEERVYYYDPTSVDAALMKRAR

RSSRDNTSLEIHPTQLSDEGEYKCRIVVWDNTGNSTEPSVPISKEARMMLKVNVPPEKVEITHLTSGRNLSLRCKSEGAK

PPADILWTKTSPDGVETEIPDLSKTREIEPAGELYDTAEQIFYQLEENEMIAKISCVVRHHNTNTTIYKNITFVSPAIQN

ALKVTIQRFAEPVLYSSIENQDGQDNKAGLVKISADPGFKVPYNTSVTLKAAAPGVFPEPRYLWWVGDQTGIAGRELRID

NLEEDKSYYVLAENPLGRVVLEVPVHITTSDYSQWNDSSRDGDVNEGDTTEGLDTGAIIGIVIGALIAVFFIGFAVWYWT

IRDGTQVFSPKKEKPHTIDEETVPFRSTEKPLLRTISTQTLEGRRSRREPSI

>SC32e allele 2

MKKAFILQVILGVLRLSDSVDLKYDDVSYATVHQSAFLRCGMMPVQPIKELWWFKIGASEERVYYYDPTSVDAALMKRAR

RSNRDNTSLEIYPTQLSDEGEYKCRIVVWDNTGNSTEPSVPISKEARMMLKVNVPPEKVEITHLTSGRNLSLRCKSEGAK

PPADILWTKTSPDGVETEIPDLAKTREIEPAGELYDTAEQIFYQLEENEMIAKISCVVRHHNTNTTIYKNITFVSPAIQN

ALKVTIQRFAEPVLYSSIENQDGQDNKAGLVKISADPGFKVPYNTSVTLKAAAPGVFPEPRYLWWVGDQTGIAGRELRID

NLEEDKSYYVLAENPLGRVVLEVPVHITISDYSQWNDSSRDGDVNEGDTTEGLDTGAIIGIVIGALIAVFFIGFAVWYWT

IRDGTQVFSPKKEKPHTIDEETVPFRSTEKPLLRTISTQTLEGRRSRREPSI

>HM9aY x 1225 F1 allele 1

MKKAFILQVILGVLRLSDSVDLEYDDVSYATVHQSAFLRCGMMPVQPIKELWWFKIGASEERVYYYDPTSVDAALMKRAR

RSSRDNTSLEICPTQLSDEGEYKCRIVVWDNTGDSTEPSVPISKEARMMLKVNVPPEKVEITHLTSGRNLSLRCKAEGAK

PPADILWTKTSPDGVETEIPDLSKTREIEPAGELYDTAEQIFYQLEENEMIAKISCVVRHHNTNTTIYKNITFVSPAIQN

ALKVTIQRFAEPVLYSSIENQDGQDNKAGLVKISADPGFKVPYNTSVTLKASAPGVFPEPRYLWWVGDQTGIAGRELHID

NLEEDKSYYVLAENPLGRVVLEVPVHITTSDYSQWNDSSRDGDVIVDDTTGGLDTGAIIGIVIGALIAVFFIGFAVWYWT

IRDGTQVFSPKKEKPHTIDEETIPFRSTDKPLLRTISTQTLEGRRSRREPSI

>HM9AY x 1225 F1 allele 2

MKKAFILQVILGVLRLSDSVDLKYDDVSYATVHQSAFLRCGMMPVQPIKELWWFKIGASEERVYYYDPTSVDAALMKRAR

RSNRDNTSLEIYPTQLSDEGEYKCRIVVWDNTGNSTEPSVPISKEARMMLKVNVPPEKVEITHLTSGRNLSLRCKSEGAK

PPADILWTKTSPDGVETEIPDLAKTREIEPAGELYDTAEQIFYQLEENEMIAKISCVVRHHNTNTTIYKNITFVSPAIQN

ALKVTIQRFAEPVLYSSIENQDGQDNKAGLVKISADPGFKVPYNTSVTLKAAAPGVFPEPRYLWWVGDQTGIAGRELRID

NLEEDKSYYVLAENPLGRVVLEVPVHITISDYSQWNDSSRDGDVNEGDTTEGLDTGAIIGIVIGALIAVFFIGFAVWYWT

IRDGTQVFSPKKEKPHTIDEETVPFRSTEKPLLRTISTQTLEGRRSRREPSI

>SC27f Allele 1

MKKAFILQVILGVLRLSDSVDLEYDDVSYATVHQSAFLRCGMMPVQPIKELWWFKIGASEERVYYYDPTSVDAALMKRAR

RSSRDNTSLEICPTQLSDEGEYKCRIVVWDNTGDSTEPSVPISKEARMMLKVNVPPEKVEITHLTSGRNLSLRCKAEGAK

PPADILWTKTSPDGVETEIPDLSKTREIEPAGELYDTAEQIFYQLEENEMIAKISCVVRHHNTNTTIYKNITFVSPAIQN

ALKVTIQRFAEPVLYSSIENQDGQDNKAGLVKISADPGFKVPYNTSVTLKASAPGVFPEPRYLWWVGDQTGIAGRELHID

NLEEDKSYYVLAENPLGRVVLEVPVHITTSDYSQWNDSSRDGDVIVDDTTGGLDTGAIIGIVIGALIAVFFIGFAVWYWT

IRDGTQVFSPKKEKPHTIDEETIPFRSTDKPLLRTISTQTLEGRRSRREPSI

>SC27f allele 2

MKKAFILQVILGVLRLSDSVDLEYDDVSYATVHQSAFLRCGMMPVQPIKELWWFKIGASEERVYYYDPTSVDAALMKRAR

RSSRDNTSLEICPTQLSDEGEYKCRIVVWDNTGDSTEPSVPISKEARMMLKVNVPPEKVEITHLTSGRNLSLRCKAEGAK

PPADILWTKTSPDGVETEIPDLSKTREIEPAGELYDTAEQIFYQLEENEMIAKISCVVRHHNTNTTIYKNITFVSPAIQN

ALKVTIQRFAEPVLYSSIENQDGQDNKAGLVKISADPGFKVPYNTSVTLKASAPGVFPEPRYLWWVGDQTGIAGRELHID

NLEEDKSYYVLAENPLGRVVLEVPVHITTSDYSQWNDSSRDGDAMVNDTTEGLDTGAIIGIVIGALIAVFFIGFAVWYWT

IRDGTHVFSPKKEKPHTIDEETIPFRSTDKPLLRTISTQTLEGRRSRREPSI

>SB1_TM_allele_1

MKKAFILQVILGVLRLSDSVDLKYDDVSYATVHQSAFLRCGMMPVQPIKELWWFKIGASEERVYYYDPTSVDAALMKRAR

RSSPDNTSLEIHPTQLSDEGEYKCRIVVWDNTGNSTEPSVPISKEARMMLKVNVPPEKVEITHLTSGRNLSLRCKSEGAK

PPADILWTKTSPDGVETEIPDLSKTREIEPAGELYDTAEQIFYQLEENEMIAKISCVVRHHNTNTTIYKNITFVSPAIQN

ALKVTIQRFAEPVLYSSIENQDGQDNKAGLVKISADPGFKVPYNTSVTLKAAAPGVFPEPRYLWWVGDQTGIAGRELRID

NLEEDKSYYVLAENPLGRVVLEV

>SB1_TM_allele_2

MKKAFILQVILGVLRLSDSVDLKYDDVSYATVHQSAFLRCGMMPVQPIKELWWFKIGASEERVYYYDPTSVDAALMKRAR

RSNRDNTSLEIYPTQLSDEGEYKCRIVVWDNTGNSTEPSVPISKEARMMLKVNVPPEKVEITHLTSGRNLSLRCKSEGAK

PPADILWTKTSPDGVETEIPDLAKTREIEPAGELYDTAEQIFYQLEENEMIAKISCVVRHHNTNTTIYKNITFVSPAIQN

ALKVTIQRFAEPVLYSSIENQDGRDNKAGLVKISADPGFKVPYNTSVTLKAAAPGVFPEPRYLWWVGDQTGIAGRELHID

NLEEDKSYYVLAENPLGRVVLEV

>SB2_TM_allele_1

MKKAFILQVILGVLRLSDSVDLEYDDVSYATVHQSAFLRCGMMPVQPIKELWWFKIGASEERVYYYDPTSVDAALMKRAR

RSSRDNTSLEICPTQLSDEGEYKCRIVVWDNTGDSTEPSVPISKEARMMLKVNVPPEKVEITHLTSGRNLSLRCKSEGAK

PPADILWTKTSPDGVETEIPDLSKTREIEPAGELYDTAEQIFYQLQENEMIAKISCVVRHHNTNTTIYKNITFVSPAIQN

ALKVTIQRFAEPVLYSSIENQDGQDNKAGLVKISADPGFKVPYNTSVTLKASAPGIFPEPRYLWWVGDQTGIAGRELHID

NLEEDKSYYVLAENPLGRVVLEV

>SB2_TM_allele_2

MKKAFILQVILGVLRLSDSVDLKYDDVSYATVHQSAFLRCGMMPVQPIKELWWFKIGASEERVYYYDPTSVDAALMKRAR

RSSRDNTSLEIYPTQLSDEGEYKCRIVVWDNTGNSTEPSVPISKEARMMLKVNVPPEKVEITHLTSGRNLSLRCKSEGAK

PPADILWTKTSPDGVETEIPDLSKTREIEPAGELYDTAEQIFYQLQENEMIAKISCVVRHHNTNTTIYKNITFVSPAIQN

ALKVTIQRFAEPVLYSSIENQDGQDNKAGLVKISADPGFKVPYNTSVTLKASAPGIFPEPRYLWWVGDQTGIAGRELHID

NLEEDKSYYVLAENPLGRVVLEV

>SB3_TM_allele_1

MKKAFILQVILGVLRLSDSVDLEYDDVSYATVHQSAFLRCGMMPVQPIKELWWFKIGASEERVYYYDPTSVDAALMKRAR

RSSRDNTSLEICPTQLSDEGEYKCRIVVWDNTGDSTEPSVPISKEARMMLKVNVPPEKVEITHLTSGRNLSLRCKSEGAK

PPADILWTKTSPDGVETEIPDLSKTREIEPAGELYDTAEQIFYQLQENEMIAKISCVVRHHNTNTTIYKNITFVSPAIQN

ALKVTIQRFAEPVLYSSIENQDGQDNKAGLVKISADPGFKVPYNTSVTLKASAPGIFPEPRYLWWVGDQTGIAGRELHID

NLEEDKSYYVLAENPLGRVVLEV

>SB3_TM_allele_2

MKKAFILQVILGVLRLSDSVDLKYDDVSYATVHQSAFLRCGMMPVQPIKELWWFKIGASEERVYYYDPTSVDAALMKRAK

RSSRDNTSLEIHPTHLSDEVEYKCRIVVWDNTGNSTEPSVPISKEARMMLKVNVPPEKVEITHLTSGRNLSLRCKSEGAK

PPADILWTKTSPDGVETEIPDLSKTREIEPAGELYDTAEQIFYQLEENEMIAKISCVVRHHNTNTTIYKNITFVSPAIQN

ALKVTIQRFAEPVLYSSIENQGGQDSKAGLVKISADPGFKVPYNTSVTLKASAPGVFPEPRYLWWVGDQTGIAGRELRID

NLEEDRSYYVLAENPLGRVVLEV

>SB4_TM_allele_1

MKKAFILQVILGVLRLSDSVDLKYDDVSYATVHQSAFLRCGMMPVQPIKELWWFKIGASEERVYYYDPTSVDAALMKRAR

RSSRDNTSLEIYPTQLSDEGEYKCRIVVWDNTGNSTEPSVPISKEARMMLKVNVPPEKVEITHLTSGRNLSLRCKSEGAK

PPADILWTKTSPDGVETEIPDLSKTREIEPAGELYDTAEQIFYQLQENEMIAKISCVVRHHNTNTTIYKNITFVSPAIQN

ALKVTIQRFAEPVLYSSIENQDGQDNKAGLVKISADPGFKVPYNTSVTLKASAPGIFPEPRYLWWVGDQTGIAGRELHID

NLEEDKSYYVLAENPLGRVVLEV

>SB4_TM_allele_2

MKKAFILQVILGVLRLSDSVDLKYDDVSYATVHQSAFLRCGMMPVQPIKELWWFKIGASEERVYYYDPTSVDAALMKRAK

RSSRDNTSLEIHPTHLSDEVEYKCRIVVWDNTGNSTEPSVPISKEARMMLKVNVPPEKVEITHLTSGRNLSLRCKSEGAK

PPADILWTKTSPDGVETEIPDLSKTREIEPAGELYDTAEQIFYQLEENEMIAKISCVVRHHNTNTTIYKNITFVSPAIQN

ALKVTIQRFAEPVLYSSIENQGGQDSKAGLVKISADPGFKVPYNTSVTLKASAPGVFPEPRYLWWVGDQTGIAGRELRID

NLEEDRSYYVLAENPLGRVVLEV

>SB6_TM_allele_1

MKKAFILQVILGVLRLSDSVDLKYDDVSYATVHQSAFLRCGMMPVQPIKELWWFKIGASEERVYYYDPTSVDAALMKRAR

RSSPDNTSLEIHPTQLSDEGEYKCRIVVWDNTGNSTEPSVPISKEARMMLKVNVPPEKVEITHLTSGRNLSLRCKSEGAK

PPADILWTKTSPDGVETEIPDLSKTREIEPAGELYDTAEQIFYQLEENEMIAKISCVVRHHNTNTTIYKNITFVSPAIQN

ALKVTIQRFAEPVLYSSIENQDGQDNKAGLVKISADPGFKVPYNTSVTLKAAAPGVFPEPRYLWWVGDQTGIAGRELRID

NLEEDKSYYVLAENPLGRVVLEV

>SB6_TM_allele_2

MKKAFILQVILGVLRLSDSVDLKYDDVSYATVHQSAFLRCGMMPVQPIKELWWFKIGASEERVYYYDPTSVDAALMKRAR

RSNRDNTSLEIYPTQLSDEGEYKCRIVVWDNTGNSTEPSVPISKEARMMLNVNVPPEKVEITHLTSGRNLSLRCKSEGAK

PPADILWTKTSPDGVETEIPDLSKTREIEPAGELYDTAEQIFYQLEENEMIAKISCVVRHHNTNTTIYKNITFVSPAIQN

ALKVTIQRFAEPVLYSSIENQGGQDSKAGLVKISADPGFKVPYNTSVTLKASAPGVFPEPRYLWWVGDQTGIAGRELRID

NLEEDRSYYVLAENPLGRVVLEV

**B. Sequence of the intergenic region between** ***fuhc*^sec^ and *fuhc*^tm^**

***>***Intergenic region between *fuhc*^sec^ and *fuhc*^tm^

GTACTGGCGTGTTTATTTTTTGTTGTGGACGGAACGATATTTGCAATAATTATCTCAGATAAATGTGGGTGTAATTTGAT

TAGCGTTTGTCTTGGCCGGGCAGGTGCTGAATCATATCGTCTGTTATTTAGTTTGCTCTACTGGGAGAGTAAACCCGTCG

ACGCCCACACTCATAGTAGGCACACATTTAGTTAAATATTAACTAAATAATTTCTAAAGTTTCCGTC
